# Metal Oxide Nanoparticles as Efficient Nanocarriers for Targeted Cancer Therapy: Addressing Chemotherapy-Induced Disabilities

**DOI:** 10.3390/cancers16244234

**Published:** 2024-12-19

**Authors:** Mohamed Taha Yassin, Fatimah O. Al-Otibi, Sarah A. Al-Sahli, Mohammad S. El-Wetidy, Sara Mohamed

**Affiliations:** 1 Department of Botany and Microbiology, College of Science, King Saud University, Riyadh 11451, Saudi Arabia; falotibi@ksu.edu.sa (F.O.A.-O.); 445205521@student.ksu.edu.sa (S.A.A.-S.); 2 King Salman Center for Disability Research, Riyadh 11614, Saudi Arabia; 3 College of Medicine Research Center, King Saud University, Riyadh 11451, Saudi Arabia; melwatidy@ksu.edu.sa; 4 Botany and Microbiology Department, Faculty of Science, Benha University, Benha 13511, Egypt; sara.m.elsayed@gmail.com

**Keywords:** nanocarriers, metal oxide nanoparticles, chemotherapy, targeted delivery, drug delivery, disability

## Abstract

Cancer remains a predominant global health concern, necessitating effective treatment options. Conventional cancer therapies, particularly chemotherapy, often face constraints such as low selectivity, insufficient solubility, and multidrug resistance (MDR), which diminish effectiveness and exacerbate negative effects. Metal oxide nanoparticles (MONPs), such as iron oxide, zinc oxide, and copper oxide, offer a promising solution by enhancing targeted drug delivery, reducing systemic toxicity, and mitigating chemotherapy-induced disabilities like neurotoxicity and cardiotoxicity. Nanocarriers conjugated with drugs can improve drug delivery within the body and enhance their circulation in the bloodstream. Recent advancements in MONP synthesis and functionalization have further improved their stability and drug-loading capacity, making them a valuable tool in cancer treatment. MONPs have distinctive physicochemical characteristics, enabling better imaging, drug encapsulation, and targeted medication delivery to cancerous cells. These nanocarriers enhance treatment effectiveness through focused and controlled drug release, reducing off-target effects and addressing drug resistance. This review aims to explore the potential of MONPs as efficient nanocarriers for anticancer drugs, addressing limitations of traditional chemotherapy such as poor specificity, systemic toxicity, and drug resistance. Additionally, the review discusses recent advancements in MONP synthesis and functionalization, which enhance their stability, drug-loading capacity, and compatibility.

## Simple Summary

This research reviews the potential of metal oxide nanoparticles (MONPs) for targeted cancer treatment, addressing the limitations of traditional chemotherapy, such as low selectivity, drug resistance, and toxicity. MONPs like titanium, iron, zinc, and copper oxides show promise in enhancing drug delivery and imaging, enabling focused drug release and improved therapeutic outcomes. However, challenges remain in production scalability, regulatory hurdles, and ensuring biocompatibility and safety. Continued advancements in nanoparticle engineering may bring MONP-based drug delivery closer to being used in effective clinical applications, potentially transforming cancer treatment.

## 1. Introduction

Cancer comprises a collection of disorders characterized by abnormal cell proliferation, which may metastasize to other regions of the body. Cancer-related mortality ranks second globally, behind deaths attributed to cardiovascular illnesses, and is regarded as a paramount global health concern [1]. Moreover, cancer and its therapies may result in both transient and enduring disabilities stemming from physical, cognitive, and emotional effects [2]. Tumors and surgical procedures may hinder movement, but chemotherapy and radiation can induce side effects such as neuropathy, exhaustion, and cognitive impairments, sometimes referred to as “chemo brain”. These impacts may restrict everyday activities, diminish employment capacity, and exacerbate mental health issues. The social stigma associated with apparent alterations or restrictions might result in isolation, necessitating rehabilitation or adaptive assistance for many survivors to achieve long-term recovery [3].

**Figure 1:**
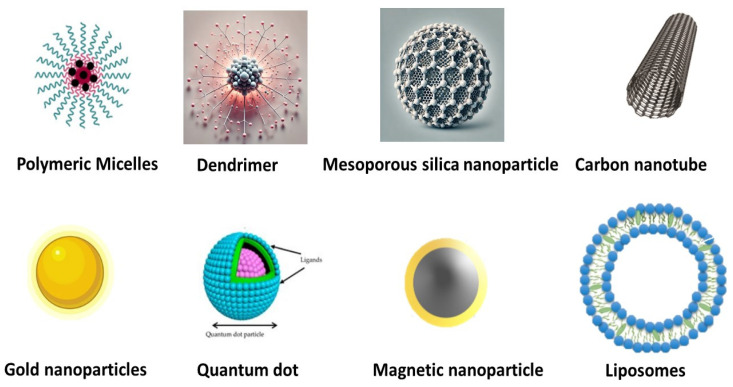
Different types of nanocarriers are used for the targeted therapy of cancer.

As a result of increasing demand, cancer treatment has evolved into a collaborative discourse among healthcare practitioners and researchers. The efficacy of treatment is contingent upon the kind of cancer, the location of the malignant tumor, and its stage of progression. Diverse techniques, such as immunotherapy, surgery, hormone therapy, chemotherapy, and radiation, are used in cancer treatment. Among them, chemotherapy is regarded as the primary option for the elimination of malignant cells [4]. However, the traditional approach to chemotherapy administration poses several challenges, including the possible development of multidrug resistance, which undermines the consistent efficacy of these medications [5]. Moreover, the conventional chemotherapy delivery methods have other limitations, including the toxic effects of chemotherapeutic chemicals on rapidly proliferating healthy cells, as well as adverse effects such as nausea, vomiting, exhaustion, hair loss, and, in severe instances, mortality [6]. Nanomedicine is an emerging discipline that has garnered considerable global interest. This technique facilitates the in-depth investigation of cellular compartments and may aid in the fight against several illnesses, including cancer [7]. The advancement of targeted therapy using nanotechnology is a significant challenge for research teams globally, and there is a substantial need for a system that integrates active functional pharmaceuticals with an effective delivery vehicle [8]. Numerous innovative nanocarriers are now used to provide anticancer agents to the targeted sites. In this context, Figure 1 depicts a structural representation of several nanocarrier-based delivery systems.

Compared to their bulk counterparts, metal oxide nanoparticles have superior physicochemical characteristics that are potentially fatal to cancer cells, rendering them advantageous for cancer medication delivery and treatment [9]. Metal oxide nanoparticles possess significant intrinsic features that are advantageous for cancer detection and imaging [10]. Superparamagnetic metal oxide nanoparticles (Fe, Mn, Gd) are promising candidates for MRI diagnostic agents [11]. Iron oxide nanoparticles can simultaneously direct cancer therapeutics to tumor cells, enabling precise administration and imaging [12]. Gold and silver nanoparticles demonstrate surface plasmonic resonance, endowing them with imaging and tumoricidal capabilities [13]. Moreover, metal oxide nanoparticles have the capability to encapsulate, solubilize, and bind to several cancer therapies, including proteins, peptides, dendrimers, polymers, antibodies, as well as hydrophobic and hydrophilic medicines [14]. The binding of medicinal drugs to or inside nanoparticles addresses several delivery challenges, including stability, solubility, and other issues, hence increasing their pharmacokinetic features [15]. ZnO, a wide-bandgap semiconductor, facilitates reactive oxygen species (ROS) production, biosensing, and bioimaging and exhibits specific cytotoxicity toward cancer cells [16]. It is beneficial in PDT treatment for malignant tumors. ZnO nanoparticles stimulate caspase-3 enzyme activity, DNA breakage, and cellular oxidative stress in cancer cells [17]. Conventional chemotherapeutic drugs, while effective, have serious side effects and impairments, such as neurotoxicity, cardiotoxicity, nephrotoxicity, and myelosuppression. These adverse effects result from the nonspecific targeting of rapidly proliferating cells, which affects healthy tissues and causes long-term consequences such as peripheral neuropathy, cognitive deficits, and chronic tiredness. Addressing these issues requires new delivery strategies that reduce systemic toxicity [18]. Metal oxide nanoparticles, such as ZnONPs, CuO NPs, and Fe_3_O_4_ NPs, have emerged as promising drug nanocarriers. In this context, MONPs overcome the limits of traditional chemotherapy with their distinct physicochemical features. Their high surface area, biocompatibility, and ease of functionalization enable targeted delivery and controlled drug release. Functionalized MONPs can selectively accumulate in tumors, reducing off-target effects. This targeted approach enhances therapeutic efficacy while allowing for lower doses of chemotherapeutic agents, significantly reducing dose-dependent toxicities [19]. They provide targeted medication administration by modifying their surfaces to attach preferentially to tumor cells, boosting specificity and lowering systemic toxicity. Their capacity to promote medication solubility and stability improves therapeutic effectiveness at lower dosages while reducing unwanted effects. MONPs can also overcome MDR mechanisms, including efflux pumps, by allowing intracellular drug transport and regulating release. These characteristics make MONPs an attractive platform for overcoming the issues of low specificity, systemic toxicity, and MDR in chemotherapy.

The use of MONPs has garnered significant attention, especially in medicine, since it enhances the therapeutic index of drugs via site-specific targeting, mitigates multidrug resistance, and facilitates the efficient delivery of therapeutic agents. Besides medicinal administration, nanocarriers are valued in both in vivo and in vitro diagnostics, improved nutraceutical formulations, and the formation of materials with efficient biocompatibility properties. Metallic nanocarriers in drug delivery systems present substantial advantages, such as extended circulation time, increased stability, essential pharmacokinetic distribution, and the ability to target sites selectively through both passive and active mechanisms. Delivering therapeutics effectively to the target site is crucial for tackling the widespread global challenge of cancer cell resistance to multiple chemotherapeutic drugs. Metal-based nanocarriers have enhanced efficacy in delivering anticancer drugs to target organs, hence reducing the previously required dosage owing to their inherent anticancer qualities. Certain metal oxides, notably IONPs, ZnO NPs, and CuO NPs, have been applied in the targeting, thermal therapy, and imaging of breast cancer cells, as well as other types of cancer cells. The worldwide interest in developing innovative nanocarriers for targeted cancer treatment needs a greater emphasis on various kinds of effective nanocarriers. This work will focus on the importance of MONPs as efficient nanocarriers for the targeted therapy of cancer, their method of synthesis, and their mode of action as nanocarriers.

## 2. Advantages of Nanocarriers in Drug Delivery

Cancer remains a leading cause of death globally, with chemotherapy a cornerstone treatment that is often hindered by challenges such as drug resistance, poor water solubility, and limited selectivity. These limitations also impede efficient drug delivery to tumor sites, reducing therapeutic efficacy. Nanoparticles (NPs) in drug delivery systems (DDS) offer a solution by encapsulating hydrophobic drugs within micelles, enhancing solubility and targeted delivery. Dendrimers, with their versatile binding sites, support the incorporation of both hydrophobic and hydrophilic drugs. Liposomes, chitosan-based nanoparticles, and cyclodextrins are particularly effective in addressing solubility and bioavailability challenges [20]. Liposome-mediated drug delivery ensures active compounds are transported directly to targeted sites, improving cancer therapy outcomes. Additionally, NP-based approaches counteract drug resistance, such as P-glycoprotein-mediated resistance, demonstrating significant potential in advancing cancer treatment strategies [21].

Nanotherapeutic drug delivery systems (NDDSs) offer several technical advantages, including the ability to incorporate both hydrophilic and hydrophobic drugs and improved shelf life. They can be administered via various routes, such as oral, nasal, parenteral, and intraocular, enhancing flexibility in treatment. NDDSs improve the biodistribution of oncological drugs, with optimal nanoparticle dimensions and surface properties prolonging circulation time and enabling controlled, sustained drug release during transit and at target sites. Additionally, NDDSs boost intracellular drug concentration through mechanisms like enhanced permeability and retention (EPR) or endocytosis, addressing challenges like multidrug resistance, poor selectivity, and low water solubility that arise in conventional cancer therapies. Targeted therapy aims to deliver chemotherapeutics directly to cancer cells, minimizing the collateral damage to rapidly dividing normal cells and reducing adverse effects. This precision enhances the efficacy of cancer treatments while mitigating the limitations of traditional approaches.

Metal oxide nanoparticles improve pharmacokinetics and biodistribution via many methods. Surface functionalization utilizing biocompatible polymers such as polyethylene glycol (PEG) [22], dextran [23], or poly(lactic-co-glycolic acid) (PLGA) [24] induces a “stealth effect”, diminishing opsonization by the reticuloendothelial system (RES) and prolonging circulation time, thereby enhancing biodistribution and retention in target tissues. External magnetic field targeting facilitates accurate delivery, as evidenced by Park et al., wherein PLGA-coated IONPs exhibited substantial deposition in xenograft models under high-gradient magnetic guiding [25]. Moreover, IONPs utilize the increased permeability and retention (EPR) effect to passively aggregate in tumors, preferentially concentrating in tumor tissues while preserving healthy ones, as has been demonstrated with PEGylated IONPs [26]. Receptor-mediated endocytosis enhances selectivity by functionalizing MONPs with ligands such as folic acid or chlorotoxin; for instance, Veiseh et al. achieved selective accumulation of chlorotoxin-conjugated IONPs in brain tumors [27]. Furthermore, polymeric coverings like PLGA facilitate regulated medication release, improving therapeutic effectiveness and reducing systemic adverse effects [28].

## 3. Nanocarriers for Targeted Therapy

**Figure 2:**
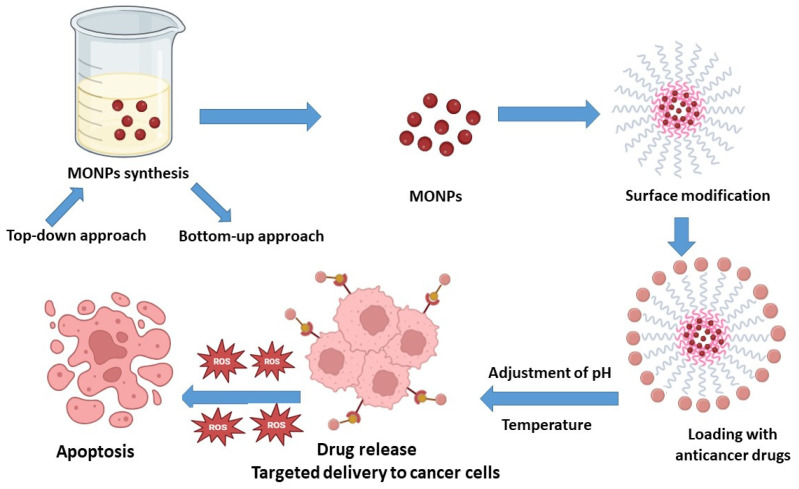
Diagrammatic illustration of the critical steps in using metal oxide nanoparticles for targeted cancer treatment. Each step delineates a phase in the process, including synthesis, surface functionalization, drug loading, targeted delivery, cellular uptake, and finally, the therapeutic impact.

Active targeting involves nanoparticles (NPs) binding to ligand receptors at the tumor site, facilitated by close proximity interactions (~0.5 nm) [29]. Functionalization with agents such as proteins, peptides, aptamers, and polysaccharides enhances tumor-specific delivery [30]. Biodegradable polymers, liposomes, dendrimers, nanoshells, and nucleic acid-based nanoparticles are widely used for targeted cancer therapy due to their biocompatibility and stability [31], ensuring therapeutic concentrations over extended periods [32]. Effective targeting requires sustained, site-specific drug release, which can be controlled via diffusion, swelling, mass transfer, or erosion [33]. Modifying polymers further refine release rates, improving therapeutic outcomes and patient compliance [34]. Targeted treatment involves precise drug delivery with sustained release to the tumor site (Figure 2) [35].

### 3.1. Strategies for Ensuring Safe Delivery of Targeted Drugs in Cancer Therapy

#### 3.1.1. Overcoming Lack of Selectivity with Nanoparticle Drug Delivery Systems

Nanoparticle drug delivery systems can improve the selectivity of anticancer drugs through mechanisms such as active targeting, passive targeting, transferrin-mediated targeting, and folate-mediated targeting [36].

#### 3.1.2. Combating Multidrug Resistance Using Nanoparticle Drug Delivery Systems

Multidrug resistance (MDR) is a major challenge in cancer treatment, with some cancers (e.g., lung and rectal) exhibiting intrinsic resistance from the outset [37]. Resistance mechanisms involve proteins in the cell membrane, cytoplasm, and nucleus, including (a) glutathione detoxification enzymes, (b) transmembrane efflux proteins, and (c) alterations in apoptosis regulators like p53 and Bcl-2. P-glycoprotein (Pgp), the key efflux pump, uses ATP to expel anticancer agents from cells [38]. To overcome MDR, NDDSs incorporate chemosensitizers, solid lipid nanoparticles, mesoporous silica nanoparticles, poloxamers, polymeric nanoparticles, and magnetic nanoparticles, enhancing therapeutic efficacy [39,40].

#### 3.1.3. Improving Aqueous Solubility with Nanoparticle Drug Delivery Systems

Pharmaceutical compounds are considered highly soluble when their maximum dosage dissolves in less than 250 mL of water within the physiological pH range of 1 to 8; however, many anticancer agents have poor aqueous solubility [41]. Poor solubility leads to reduced bioavailability, increased dietary interactions, incomplete drug release, and greater interpatient variability [42]. To overcome these challenges, two main strategies are employed: (a) enhancing saturation solubility through complex formation and (b) increasing dissolution rate using techniques such as nanonization or using nanocrystals [43], albumin-based nanoparticles, liposomal formulations [44], polymeric micelles [45], cyclodextrin-based nanoparticles [46], or chitosan-based nanoparticles [47].

## 4. The Synthesis of Metal Oxide Nanocarriers

**Figure 3:**
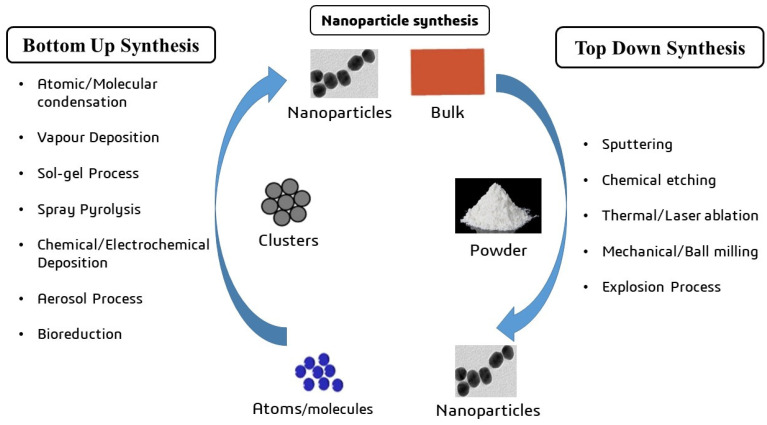
Synthesis of the metal oxide nanoparticles through bottom-up and top-down approaches.

To produce effective nanocarriers for drug delivery, nanoparticles (NPs) need to possess an appropriate particle size [48], uniform dispersion, and high drug-loading capacity [49]. Metal oxide NPs enhance the delivery and efficacy of pharmaceuticals by penetrating intracellular endocytic channels and targeting specific sites [50]. The synthesis of a nanocarrier for medication delivery typically involves two main approaches: the top-down and bottom-up methods (Figure 3). The top-down method fragments metal oxide materials into smaller particles using processes like laser ablation and thermal disintegration [51], while the bottom-up method builds nanoparticles from atoms, such as through chemical vapor manufacturing using spinning laser pyrolysis [52]. Metal oxide nanoparticles are combined with specific polymer molecules to create efficient and durable nanocarriers, encapsulating the drug for targeted delivery to the site of action [53]. However, these nanoparticles often lack the surface functional groups necessary for biological applications. Surface functionalization is crucial for enhancing biocompatibility and water dispersibility and providing appropriate surface groups for conjugation [54]. An example of a novel approach involves a core–shell pH-responsive nanocarrier with a magnetic nanoparticle core modified with hyperbranched polyglycerol. The magnetic core is coated with doxorubicin and encapsulated in a secondary shell of PEGylated carboxymethylcellulose, cross-linked using borax to assess drug-loading capacity and pH responsiveness [53].

Recent advancements in the scalable production of MONPs have emphasized cost-effectiveness and sustainability. Green synthesis techniques, including the use of plant extracts and microbial systems, provide environmentally sustainable and economically viable alternatives by minimizing the use of harmful solvents and energy requirements [55]. Continuous flow reactors provide high-throughput manufacturing with meticulous control over reaction conditions, assuring repeatability and minimizing batch variability [56]. Other methods, such as spray pyrolysis and thermal decomposition, have been refined for industrial-scale manufacturing, providing consistent particle size while minimizing energy expenses [57]. Improvements in sol–gel and hydrothermal techniques have increased scalability by reducing waste and optimizing precursor use [58], whilst supercritical fluid technology offers an environmentally friendly option for accurate nanoparticle synthesis devoid of toxic solvents [59].

## 5. Targeted Drug Delivery Systems for Cancer Cells

Targeted drug delivery systems offer a novel approach to effectively administering pharmaceuticals to cancer cells, with benefits including safeguarding normal cells from pharmacological effects, reducing adverse medication reactions, and lowering drug resistance rates in some cancer cells [60]. It is crucial for drugs to reach their target location in adequate amounts to be effective [61]. The cell nucleus is the principal target for many drugs, but despite the design of nanoparticles to facilitate entry into the cytoplasm, there is no guarantee they will successfully access the nucleus [62].

### 5.1. Active Targeting

**Figure 4:**
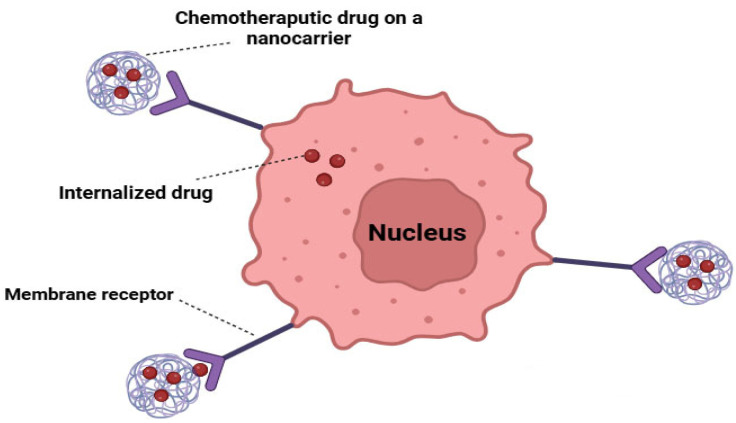
Active targeted therapy of cancer using chemotherapeutic drugs loaded on nanocarriers.

Active drug targeting utilizes molecular structures such as antibodies or peptides to reach specific receptors and target areas (Figure 4) [63]. It involves three key components: a ligand functioning as a targeting moiety, a polymer serving as a carrier, and the specific pharmaceutical of interest [64]. The antigen acts as an active targeting mechanism, expressed exclusively on cancer cells, enabling the internalization of the system through receptor-mediated endocytosis. This approach enhances the precision of drug delivery to cancer cells [65].

Recent research has shown that particular MONPs can greatly increase tumor penetration and retention compared to other nanocarriers. For instance, PEGylated IONPs have been found to boost tumor targeting and retention via both the enhanced permeability and retention impact and external magnetic field guiding, resulting in better treatment effects in vivo [25]. Moreover, curcumin is renowned for its capacity to eradicate cancer cells, yet its inadequate water solubility and limited absorption provide considerable obstacles to its therapeutic use. A study was conducted to develop a curcumin-loaded poly(methyl methacrylate) (PMMA) and poly(ethylene glycol) (PEG) ZnO bio-nanocomposite to enhance the bioavailability and effectiveness of curcumin. PEG and PMMA are appropriate for application in controlled-release polymer systems owing to their established safety. The synthesized nanocomposite has been shown to effectively transport a significant quantity of curcumin, facilitating the quick release of its therapeutic payload at a low pH, hence improving curcumin bioavailability and anticancer efficacy against stomach cancer (AGS) cells [66]. Furthermore, cerium oxide nanoparticles (CeO_2_ NPs) have exhibited improved medication retention and pharmacokinetics in tumors, benefiting from their antioxidant characteristics [67].

### 5.2. Passive Targeting

One common example of passive drug targeting is the direct injection of a drug into the bloodstream to reduce elimination by processes such as phagocytosis, excretion, opsonization, and metabolism [68]. Under certain conditions like inflammation and hypoxia, blood vessels become more permeable [69], particularly when tumor cells initiate angiogenesis, leading to the formation of new blood vessels. This increased permeability allows the passage of macromolecules, including nanosystems, facilitating the delivery of encapsulated pharmaceuticals in nano form. This form of targeting relies on the use of nanocarriers to enhance pharmacokinetics and reduce adverse effects [70].

## 6. Titanium Dioxide-Based Nanocarrier

Titanium dioxide (TiO_2_) is a naturally abundant material with key properties such as biocompatibility, low weight, high corrosion resistance, excellent thermal stability, minimal ion release, and non-magnetic behavior [71]. Accordingly, TiO_2_ nanoparticles are widely used in medical applications, particularly in cancer research. They are used in medication delivery systems, sonodynamic therapy (SDT), and photodynamic therapy (PDT) [72]. Moreover, TiO_2_ nanocarriers provide a promising option for drug delivery and therapy. In this context, it was determined that the incorporation of doxorubicin (DOX) onto TiO_2_ nanoparticles applied to breast cancer cells in a laboratory setting enhanced the drug’s anticancer activity. When TiO_2_ nanocarriers were coated with polyethylene glycol (PEG) and loaded with DOX, they enabled controlled drug release and improved DOX’s effectiveness as a chemotherapeutic agent in mice with orthotopic breast tumors, highlighting the increased efficacy of doxorubicin in vivo with TiO_2_-based nanocarriers [73]. The resilience of tumor cells is a critical component in the failure of chemotherapy in cancer patients. A drug delivery technique using mesoporous titanium dioxide nanoparticles (MTN) has been created to target both CD44 and N-cadherin in order to prevent drug resistance. DOX was selected as the model drug. Cytotoxicity tests indicated that ADH-1-HA-MTN/DOX exhibited more toxicity to tumor cells compared to its non-ADH-1 modified counterparts [74]. Table 1 outlines previous studies in the literature that utilized the TiO_2_ nanocarriers for drug delivery targeted therapy of cancer.

## 7. Metal Oxide Nanocarriers for Targeted Cancer Therapy

Metal oxide nanocarriers are essential for augmenting the release and effectiveness of anticancer medications by boosting drug stability, facilitating regulated release, and enabling targeted delivery to cancer cells [81]. These carriers often lack inherent therapeutic properties; rather, they function as vehicles to encapsulate and release anticancer drugs in a regulated way [82]. The principal benefit of employing metal oxide nanocarriers is their capacity to safeguard the encapsulated anticancer drug against fast metabolism, degradation, and elimination by the body, thereby enhancing bioavailability and minimizing systemic toxicity [83]. For example, iron oxide nanocarriers have demonstrated potential in delivering medications such as doxorubicin, which facilitates targeted delivery to tumor cells by magnetic targeting, therefore improving treatment efficacy [84]. Curcumin-loaded PMMA-PEG/ZnO nanocarriers have been studied for their ability to encapsulate curcumin, providing better control over the release rate and increasing its anticancer efficacy. This approach enhances bioavailability and reduces systemic toxicity [66]. However, many metal oxides possess intrinsic therapeutic properties. For example, ZnONPs and CuONPs generate ROS that induce cancer cell apoptosis [85]. They also enhance the cytotoxicity of chemotherapy drugs by disrupting cellular environments, such as inducing oxidative stress or altering ion homeostasis [86]. Furthermore, metal oxides may exhibit antibacterial or anti-inflammatory activity, mitigating complications during treatment. The ratio of metal oxides in nanoparticles differs according to design and use. Metal oxides may form the core (e.g., Fe_3_O_4_ in core–shell configurations) or be included inside a polymer or lipid matrix, generally comprising 10–50% by weight [87,88].

### 7.1. Iron Oxide-Based Nanocarrier

**Figure 5:**
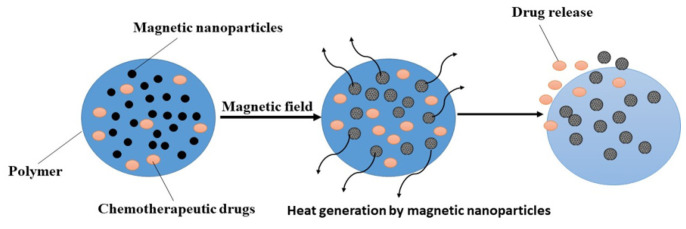
Magnetic nanoparticles and chemotherapy agents are administered together in a polymer coating. Upon reaching the cancer site, magnetic nanoparticles are heated by an external magnetic field, removing the polymer coating and releasing chemotherapeutic drugs to the tumor site.

**Figure 6:**
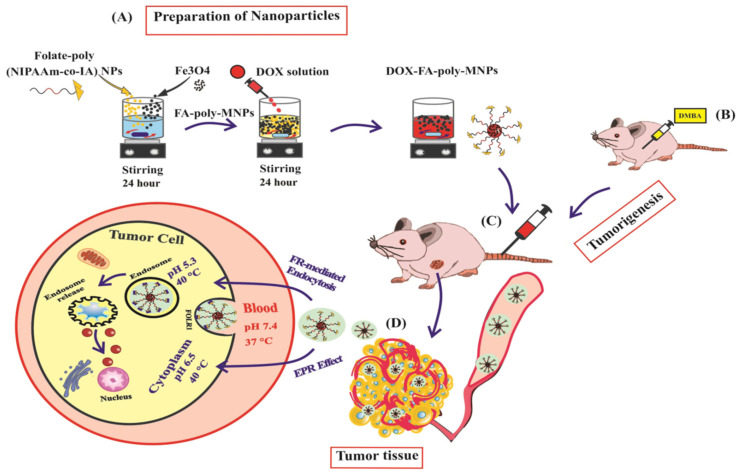
(**A**) DOX was loaded into folate-conjugated dual-responsive magnetic nanoparticles as described. (**B**) Female Sprague Dawley rats were subcutaneously injected with DMBA at 45–48 days to induce breast tumor. (**C**) The experiment began after the tumor volume reached 1000 mm^3^. The breast cancer model was given DOX-FA-poly-MNPs at a dosage of 2 mg/kg/48 h. (**D**) Folate-targeted nanoparticles aggregate at tumor locations via the increased EPR effect, also known as passive targeting. Tumor-targeted nanoparticles attach to cell surface folate receptor-α (FOLR1) via FRs-mediated endocytosis, boosting their entrance into tumor cells, a process known as active targeting. Copyright @ 2022 Pourradi et al. [92].

Magnetic iron oxide-based polymeric nanocomposites have become essential drug carriers in cancer treatment [89]. Key features of iron nanocomposites include a large surface area, superparamagnetic properties, low toxicity, and ease of separation using magnetic fields [90]. Iron oxide occurs in nature as magnetite (Fe_3_O_4_), hematite (α-Fe_2_O_3_), and maghemite (γ-Fe_2_O_3_). Iron oxide nanoparticles (IONPs) serve as the core of the ultimate configurations of therapeutic nanovectors. The primary aim in the synthesis of magnetic nanoparticles, from a physicochemical perspective, is to meticulously regulate particle size and ensure colloidal stability and dispersibility under physiological settings. The characteristics may be altered by coating the particles in two distinct manners: either by physically embedding the iron oxide nanoparticles inside a polymer matrix or by functionalizing their surface with polymer molecules [91]. Figure 5 shows the drug release of chemotherapeutic agents trapped inside a polymer with magnetic nanoparticles under the influence of a magnetic field. A prior study demonstrated the targeted administration of doxorubicin using thermo/pH-responsive magnetic nanoparticles in a rat model of breast cancer (Figure 6) [92]. The magnetic IONPs utilize several pathways for efficient, targeted therapy for cancer through magnetically guided drug delivery, vectorized magnetic nanocarriers, and MRI-guided drug delivery.

#### 7.1.1. Magnetically Guided Drug Delivery

Utilizing the magnetic characteristics of IONs, targeted medication administration may be accomplished by directing the IONs with a localized external magnetic field. This method has shown efficacy in the accumulation of nanoparticles in certain diseases, including tumors and inflammatory regions [93]. Previous studies have examined this site-directed application, which generates magnetic nanocarriers with unique structural characteristics. A prior work documented the synthesis of gold-coated iron oxide nanoparticles encapsulating the cisplatin anticancer agent. This study included the synthesis of IONs by coprecipitation, followed by oxidation to yield maghemite. The nanoparticles were then coated with gold via a technique known as ‘iterative hydroxylamine seeding’. Particle coating was then accomplished using thiolated chemicals, namely thiolated polyethylene glycol (PEG) linkers. Cisplatin was ultimately incorporated into the magnetic nanocarriers via robust coordination interactions with the PEG linker. In vitro testing revealed that these nanocarriers increased cytotoxicity by 110-fold against human ovarian cancer cell line A2780 and enabled targeted inhibition of cell proliferation when directed with a simple magnet [94]. A previous study also investigated the magnetic control of IONPs for targeted drug administration, concentrating on the formulation of PVA-coated IONPs infused with doxorubicin. Through the application of a magnetic field, these nanocarriers exhibited effective control for magnetically directed drug delivery [95].

#### 7.1.2. Vectorized Magnetic Nanocarriers

Functionalizing IONs by targeting moieties such as antibodies, peptides, or small organic compounds offers a promising approach for the targeted delivery of medicines [96]. The decision to target molecules that may engage with disease indicators via ligand–receptor or antigen–antibody interactions enables IONPs to selectively concentrate at pathological locations, hence improving their specificity and therapeutic efficacy [97]. For instance, a previous study functionalized IONPs with J591 monoclonal antibodies targeting prostate-specific membrane antigen (PSMA), which is highly overexpressed in prostate cancer. These iron oxide nanoparticles, with an inorganic diameter of 8–10 nm, were sequentially coated with β-cyclodextrin and pluronic F127 polymer, creating stable nanocarriers with docetaxel encapsulated in the hydrophobic cavity of β-cyclodextrin. In vitro, tests showed that this docetaxel-loaded nanoparticle formulation enhanced internalization in pancreatic cancer cells due to its optimal particle size and zeta potential, demonstrating anticancer effects via multiple mechanisms and highlighting its potential for targeted prostate cancer therapy [98]. In another study, researchers coated IONPs with anti-CD44 antibodies to specifically target CD44-positive pancreatic cancer cells, facilitating gemcitabine delivery to induce cell death. Disulfide linkages enabled the multifunctionalization of these IONPs, allowing controlled drug release in highly reducing environments, such as the intracellular milieu of cancer cells. As expected, these nanocarriers demonstrated targeted, rapid release under intracellular conditions and showed increased selectivity for CD44-positive pancreatic cancer cells [99].

#### 7.1.3. MRI-Guided Drug Delivery Utilizing IONs

A previous work described the creation of IONs linked with folic acid for breast cancer diagnosis and therapy. These nanocarriers were loaded with DOX and evaluated for their effectiveness in drug delivery using nude mice implanted with MCF-7 breast cancer tumors. MRI was employed to track the nanoparticles’ accumulation within the tumor site [100]. Another study focused on the formulation of DOX-encapsulated heparin-coated IONPs for integrated medication treatment and magnetic resonance imaging. These systems were distinguished by a gradual drug release, enhanced absorption efficiency relative to doxorubicin alone, and decreased cardiotoxicity [101].

#### 7.1.4. Drug Delivery Systems Activated by External Stimuli

Alternative approaches to enhance drug release at targeted locations involve the use of functional groups or stimuli-responsive coatings. One common method leverages the difference in pH between healthy and cancerous tissues. Tumors generally have a lower extracellular pH than normal tissues, which creates an opportunity to activate drug release specifically in tumor environments. This pH disparity can be exploited by employing nanocarriers equipped with pH-sensitive bonds or coatings, such as liposomes or specialized polymers, to trigger the controlled release of therapeutic agents [102]. For instance, PEGylated iron nanoparticles loaded with doxorubicin were synthesized using a method that pre-formed a DOX-Fe^2+^ complex. This complex may associate with hydroxyl groups on the nanoparticle surface and dissociate at acidic pH, leading to pH-dependent drug release [103]. Moreover, another study developed doxorubicin-loaded chitosan-coated mesoporous iron oxide nanoparticles, which showed improved therapeutic efficacy when exposed to an alternating current magnetic field (ACMF) [104]. Quinto et al. conducted a study on the synthesis of phospholipid-polyethylene glycol-coated IONPs with a 14 nm core diameter [105]. These nanocarriers generated sufficient heat to raise the temperature to 43 °C while releasing doxorubicin in a controlled manner, highlighting their potential for improved efficacy in chemotherapy–hyperthermia combination cancer treatments. Table 2 summarizes recent studies on magnetic IONP-based nanocarriers for cancer therapy.

### 7.2. Zinc Oxide-Based Nanocarrier

**Figure 7:**
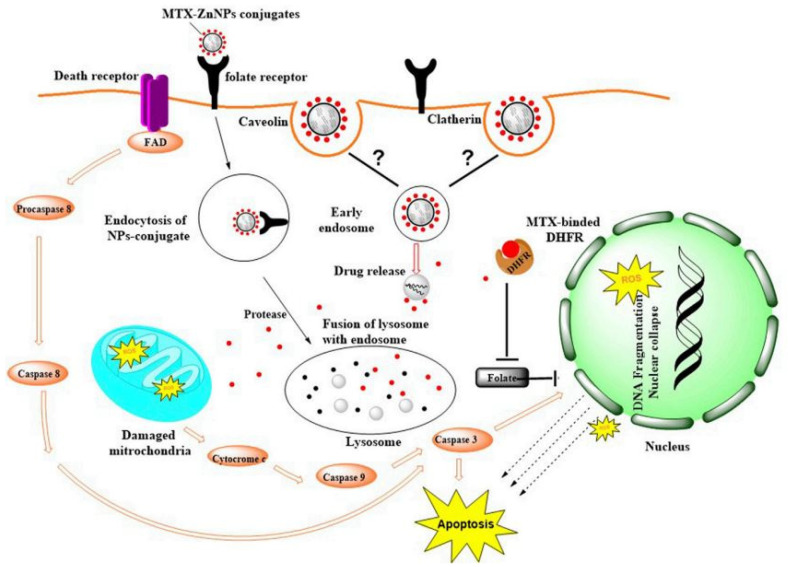
Illustration of potential MTX-ZnONP internalization through caveolin-mediated endocytosis and the mechanism of MTX action in A549 cells. Copyright @ 2023 Mishra et al. [122].

ZnO nanoparticles (ZnO NPs), similar to other nanocomposites, function as significant carriers for the administration of many anticancer and anti-inflammatory medicines [115]. ZnO is a metal oxide of great scientific interest due to its mechanical and chemical stability, making it a suitable material for pharmaceutical applications. It has been explored as a drug delivery platform for targeted treatment and minimizing side effects [115,116]. ZnONP-based drug delivery systems (DDS) offer numerous advantages, including (a) the inhibition of premature drug release in the bloodstream, thereby reducing the risk of systemic toxicity; (b) the facilitation of drug transport to target cells/organs through active targeting, thereby increasing efficacy; (c) the enhancement of aqueous solubility and improvement of the pharmacokinetics of hydrophobic drugs; and (d) minimal or negligible toxic effects on normal and healthy tissues/organs [117]. A previous study demonstrated the synthesis of a pH-sensitive nanocarrier by integrating a hydrogel nanocomposite consisting of PEG, PVA, and ZnO nanoparticles with a substantial surface area [118]. Zheng et al. (2017) engineered core–shell nanocarriers (ZnO-DOX@ZIF-8) comprising mesoporous ZnO as the core and microporous zeolitic imidazolate frameworks (ZIF-8) as the shell for the loading and pH-responsive transport of DOX. The mesoporous ZnO core serves as a drug storage reservoir, while the ZIF-8 operates as a stable shell that inhibits premature drug release at a physiological pH. Upon the internalization of ZnO-DOX@ZIF-8 by A549 cancer cells, DOX was discharged by the dissolution of ZnO and the disintegration of ZIF-8 in mildly acidic intracellular environments. Furthermore, ZnO-DOX@ZIF-8 demonstrated synergistic antitumor efficacy via ZnO-mediated reactive oxygen species production [119]. Moreover, a previous study documented the use of hyaluronic acid (HA)-functionalized ZnO quantum dots (QDs) for the pH-responsive administration of DOX in A549 cells. DOX was immobilized on the surface of ZnOQDs by the creation of a six-membered chelate involving Zn^2+^ and the oxygenated functional groups of the anthraquinone moiety in DOX. HA functionalization on ZnOQDs facilitates the identification of CD44-overexpressing A549 cells and enables drug release via the disintegration of the metal–DOX complex, resulting from the dissolution of ZnONPs in the acidic intracellular environment. Accordingly, the HA-functionalized ZnOQD-DOX exhibited enhanced cytotoxicity relative to non-targeted ZnOQD-DOX owing to increased cellular absorption [120]. Furthermore, other investigators developed a temperature- and pH-sensitive drug delivery system by conjugating ZnONPs with poly-(N-isopropylacrylamide), a thermally responsive polymer, while encapsulating DOX. The capability of nanoformulation to react at pH variations was shown by the in vitro release profile in the presence of an acetate buffer at pH 5.0 and 6.0 [121]. A previous study described that MTX-ZnONPs leverage receptor-mediated endocytosis to facilitate their internalization into cancer cells, exploiting specific cellular pathways for targeted delivery. Once inside the cells, the conjugated methotrexate (MTX) acts by inhibiting dihydrofolate reductase (DHFR), a key enzyme involved in the folate cycle. By blocking DHFR activity, MTX prevents the conversion of dihydrofolate to tetrahydrofolate, an essential cofactor for nucleotide synthesis. This disruption halts the production of purines and thymidylate, which are critical for DNA replication and repair, ultimately impairing cellular proliferation. This mechanism effectively induces cytotoxicity in A549 cells, demonstrating a promising therapeutic strategy for lung cancer treatment (Figure 7) [122]. In an earlier study, the researchers evaluated a combination of various-sized ZnONPs and daunorubicin under UV irradiation. The research indicated that this combination may have a synergistic lethal impact on K562 and adriamycin-resistant K562/A02 leukemia cells, highlighting the significant potential of ZnONPs in relevant medicinal and biological applications [123]. Dine et al. (2018) documented the use of fluorescent ZnO/copolymer core/shell quantum dots, using ZnO quantum dots as the core and a copolymer of 2-(2-methoxyethoxy) ethyl methacrylate (MEO2MA) and oligo (ethylene glycol) methacrylate (OEGMA) as the shell for pH- and thermoresponsive DOX administration. The polymeric shell may enclose the drug and release it in response to stimulation. The DOX-loaded ZnO/copolymer QDs exhibited enhanced cytotoxicity in HT29 cells relative to free DOX, attributed to their efficient cellular uptake and subsequent release of DOX into the cytosol in the presence of physiological temperature resulting from the dissolution of ZnO QDs. The augmented cytotoxicity of DOX-loaded ZnO/copolymer QDs under thermal conditions is ascribed to the heightened release of DOX due to the phase transition of the grafted copolymer from an expanded to a collapsed state [124]. Recently, Li et al. (2019) used N-acetyl-L-cysteine-linked ZnONPs to load and deliver camptothecin. Camptothecin was conjugated on the surface of ZnONP via covalent bonding. The drug-loaded nanocomposites showed stronger cytotoxicity in A549 cells than the free drug in a synergistic way [125]. Other studies have also utilized ZnONPs for the formation of a drug delivery system for the successful release of anticancer drugs, such as etoposide [126], isotretinoin [127], and daunomycin [68]. Table 3 summarizes recent studies on ZnONPs as nanocarriers for targeted cancer therapy.

### 7.3. Copper Oxide-Based Nanocarrier

**Figure 8:**
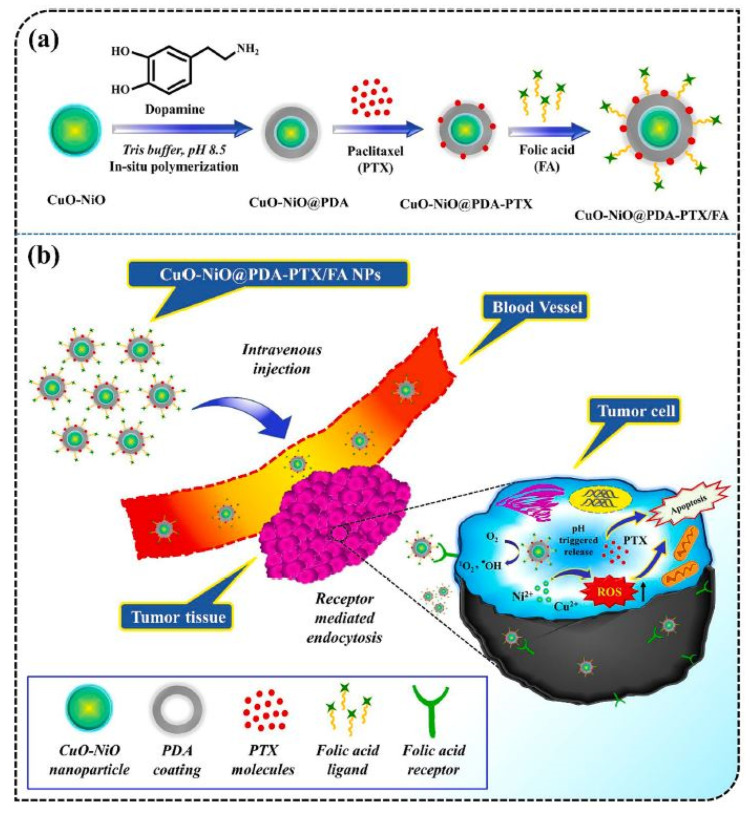
(**a**) Diagram depicting the synthesis process of the CuO-NiO@PDA-PTX/FA drug delivery system. (**b**) Conceptual illustration of the CuO-NiO@PDA-PTX/FA noncomplex, highlighting the combination of bimetallic oxide nanoparticles and a chemotherapeutic agent for enhanced breast cancer treatment. Copyright @ 2023 Singh and Pal 2023 [142].

Copper nanoparticles (CuONPs) have increasingly emerged as a prominent research focus owing to their distinctive physical, chemical, electrical, and optical characteristics, as well as their cost-effectiveness and accessibility [137]. The functionalization of the nanocarrier is important for targeted delivery to the organ, ensuring an optimal operational environment [138]. A study on drug delivery in rats was performed using CuO@BSA, a composite made of synthesized CuONPs and bovine serum albumin (BSA) in a biological medium. Curcumin (CUR), an anticancer agent, was encapsulated within the CuO@BSA composites, which acted as the nanocarrier. The release rate of the drug was 75% after 48 h at pH 7.4. Cytotoxicity tests demonstrated that CuO@BSA-CUR exhibited toxic effects on MDA-MB-231 cells, while CuO@BSA alone showed no toxicity [139]. A prior work has shown that folic acid and starch modification boosted the penetration of CuONPs into cancer cells via folate receptor-mediated endocytosis, hence improving breast cancer treatment [140]. Experimental studies aimed at advancing targeted drug delivery and bioimaging agents led to the development of transferrin (Tf)-templated copper nanoclusters (Tf-CuNCs) with enhanced luminescence properties. These nano molecules were further synthesized into spherical transferrin copper nanocluster-doxorubicin (DOX) nanoparticles (Tf-CuNC-DOX-NPs) through electrostatic interactions with doxorubicin. The newly developed nanomaterials were then tested in vivo on Tf receptor (TfR)-positive DLA (Dalton’s lymphoma ascites) tumor-bearing mice. The results demonstrated significant tumor growth inhibition and prolonged survival in the treated animals [141]. A previous study found that biocompatible starch-based polymers offer a pH-dependent drug release profile in cancerous cells. Moreover, the incorporation of folic acid into green-synthesized nanoparticles particularly targets the overexpressed folate receptors on the surface of malignant cells. This results in a mitochondria-associated apoptotic mechanism in FA-coated CuONPs inside the MDA-MB-231 breast cancer cell line [140]. Table 4 summarizes current research on CuONPs as nanocarriers for targeted delivery of anticancer drugs in cancer treatment. A prior investigation showed the biosynthesis of nanocarrier CuO-NiO@PDA-PTX/FA for the targeted delivery of paclitaxel anticancer drug to tumor cells [142]. In this study, a versatile multifunctional nanoplatform was generated through ligand modification and mussel adhesion of polydopamine. This platform enhances the targeted delivery of PTX while simultaneously sequestering Cu^2+^ and Ni^2+^ ions to achieve a synergistic effect in combating the growth of pernicious, cancerous tissues (Figure 8).

## 8. Metal Oxide Nanoparticles for Theranostic Applications

Metal oxide nanoparticles (MONPs) have advanced notably in theranostic applications, integrating therapeutic and diagnostic functions to improve cancer therapy. A significant development in this field is the use of IONPs, which have been extensively investigated for their capacity to transport pharmaceuticals while concurrently functioning as MRI contrast agents, enabling real-time observation. Peng et al. (2018) created multifunctional IONPs that contained DOX for treatment and integrated MRI-active iron oxide for imaging, facilitating the non-invasive monitoring of medication delivery to tumor locations [150]. These IONPs not only augmented the therapeutic efficiency of DOX by enhancing tumor accumulation via the increased permeability and retention effect, but also facilitated accurate monitoring of drug distribution and tumor response by MRI imaging. Moreover, ZnO NPs have potential in theranostic applications, especially in hyperthermia and imaging. Zhang et al. (2020) conducted a study investigating the capabilities of ZnO nanoparticles as a dual-function nanocarrier, wherein the nanoparticles were functionalized with imaging agents, including fluorescein, and loaded with chemotherapeutic agents. The ZnO nanoparticles facilitated real-time optical imaging for tumor localization and permitted localized heat production when subjected to an alternating magnetic field for hyperthermia therapy [151]. Furthermore, CeO_2_ NPs have been employed for theranostic applications, notably in cancer treatments, owing to their antioxidant capabilities and the essential role of oxidative stress. Liu et al. (2021) conducted research in which CeO_2_ nanoparticles were integrated with therapeutic drugs and modified for imaging purposes. The nanoparticles selectively accumulated in tumor tissues, reducing oxidative stress in adjacent normal tissues and functioning as MRI contrast agents, thus enabling in vivo imaging to monitor tumor responses to therapy [152]. This amalgamation of therapeutic and diagnostic tasks improved both treatment accuracy and monitoring efficacy.

## 9. Barriers to Effective Drug Release in Nanocarrier Systems

The use of metal oxide nanocarriers has garnered significant attention, particularly in cancer therapy, since it enhances the therapeutic efficacy of chemotherapeutic medicines via site specificity, mitigates multidrug resistance, and facilitates the efficient delivery of therapeutic agents [83]. Nonetheless, the use of nanocarriers remains nascent, with most research occurring in laboratory settings, and there is a paucity of effective clinical trials applicable to medical practice. Moreover, the fabrication of nanocarriers necessitates meticulous design and engineering, rigorous characterization of their physicochemical properties, and reproducible scaling and manufacturing processes [153]. The high costs of raw materials and the complex, multistep manufacturing processes pose significant challenges to pharmaceutical companies in scaling up the production of nanocarriers. For instance, products like Abraxane^®^ and Doxil^®^ are considerably more expensive than their non-encapsulated counterparts, paclitaxel and doxorubicin [154].

Moreover, it is challenging to determine which kind of nanoparticles is optimal for reversing cancer multidrug resistance. The tumor microenvironment may restrict nanocarriers’ penetration into the tumor because of elevated interstitial pressure, abnormal blood vessel structures, and the thick extracellular matrix [155]. In addition, the toxicity of nanocarriers, their evasion of the phagocytic system, their ability to bypass physiological barriers, and their potential to elicit an immunological response are critical concerns that must be meticulously considered before using them in live organisms [96]. Nonetheless, metal oxide nanocarriers have additional constraints, including contradictory modes of action and restricted feasibility for extensive clinical use. Moreover, the possible toxicity of metal oxide nanocarriers and their elimination from the body provide obstacles in therapeutic use [156].

Visualizing and detecting the in vivo biodistribution of nanocarriers over time is highly challenging. Soon after administration, nanocarriers or their degradable soft components enter biological fluids and may interact with these fluids (such as blood serum) or with biomolecules like proteins. These interactions can substantially alter their physicochemical properties, including size, drug-loading capacity, release profile, and potential for aggregation, ultimately affecting the functionality of nanomedicine within biological systems [157]. Finally, the regulatory frameworks for nanomedicines are still developing, and the approval process is often more protracted and intricate than that for traditional pharmaceuticals. This arises from the need for comprehensive assessments of toxicity, biodistribution, and long-term impacts.

Metal oxide nanoparticles, despite their potential, encounter several substantial hurdles when employed as nanocarriers for targeted cancer treatment. Challenges such as inadequate bioavailability, unregulated release rates, and ineffective targeting persist as significant barriers [158]. The dimensions, surface charge, and surface chemistry of these nanoparticles can change their interactions with biological systems, hence influencing their efficacy in targeting and reaching cancer cells [159]. Moreover, the possibility of cytotoxicity and the activation of immunological responses may hinder their therapeutic use [160]. Advancements in nanoparticle design are essential to address these problems, necessitating adjustments to improve stability, specificity, and biocompatibility [161]. Advanced surface functionalization methods and the creation of tailored delivery systems are crucial for enhancing therapeutic efficacy and reducing off-target effects [162].

Innovative preclinical models and regulatory pathways are being employed to tackle the difficulties of biocompatibility and toxicity associated with the use of MONPs in cancer treatment. Advanced 3D tissue models and organ-on-chip platforms offer more authentic settings for investigating the interactions and long-term consequences of MONPs than conventional 2D systems [163]. Regulatory frameworks, particularly directives from the FDA and EMA, underscore the necessity of comprehensive testing across several animal models to guarantee safety and efficacy [164]. Surface changes, including biocompatible polymer coatings and stimuli-responsive designs, enhance MONP targeting and diminish off-target toxicity [165]. Furthermore, in silico prediction models are progressively employed to mimic biological interactions, providing insights into possible dangers and informing preclinical investigations [166].

## 10. Conclusions

Nanotechnology, particularly through improved MONP-based drug delivery systems, holds great potential to transform cancer treatment by addressing many of the limitations of conventional chemotherapeutic therapies. Conventional chemotherapy often suffers from issues like poor specificity, systemic toxicity, and the emergence of MDR. Metal oxide nanocarriers, including those based on titanium, iron, zinc, and copper, demonstrate substantial promise in overcoming these challenges due to their unique physicochemical characteristics and versatile applications in targeted drug delivery. For instance, titanium dioxide and iron oxide nanoparticles not only enhance the specificity of drug targeting but also facilitate imaging and tumor tracking. Furthermore, IONPs enable magnetically guided drug delivery, while zinc oxide and copper oxide nanocarriers can be engineered to release drugs in response to specific stimuli, such as pH changes, optimizing therapeutic impact and reducing off-target effects. The primary advantage of MONPs is their capacity to provide site-specific, controlled drug release, which enhances the concentration of chemotherapeutic agents at tumor sites, minimizes systemic side effects, and effectively counters MDR mechanisms. Through ligand–receptor interactions or magnetic guidance, these nanocarriers can be directed precisely to cancer cells, sparing healthy tissue and thereby reducing the severe side effects commonly associated with chemotherapy. Additionally, the unique properties of MONPs allow them to serve as diagnostic tools, thus enabling dual therapeutic and diagnostic functions. This approach enhances the accuracy of treatment monitoring and facilitates the early detection of drug response in tumors.

Despite their potential, several hurdles impede the widespread clinical implementation of MONP-based drug delivery systems. The synthesis of these nanoparticles is complex, requiring stringent control over size, dispersion, and biocompatibility, which can result in high production costs and challenges in scalability. Moreover, the biocompatibility and long-term toxicity of MONPs remain under investigation, and the potential for unexpected immunogenic reactions necessitates comprehensive preclinical and clinical testing. Regulatory approval processes are also particularly challenging for nanomedicine, given the need for thorough assessments of the unique pharmacokinetics and biodistribution patterns associated with nanoparticles.

In conclusion, while MONPs demonstrate substantial potential as efficient and selective nanocarriers for cancer therapy, achieving a balance between efficacy, safety, and cost-effectiveness is crucial for their success in clinical applications. Ongoing advancements in nanoparticle engineering, functionalization strategies, and biocompatibility assessments are essential for translating these innovative therapeutic approaches from the laboratory to the clinic. Future research should focus on optimizing manufacturing processes, enhancing the safety profile of MONPs, and overcoming regulatory challenges. With continued interdisciplinary collaboration and rigorous testing, MONP-based drug delivery systems may indeed revolutionize cancer therapy, offering patients more effective and safer therapeutic options.

Innovative advances in the use of MONPs for therapeutic applications, including AI-driven design and machine learning, are markedly progressing in the sector. Artificial intelligence and machine learning are revolutionizing the design process by forecasting material characteristics, enhancing surface alterations, and refining the targeting of cancer cells. These technologies provide the swift assessment of nanoparticle interactions with biological systems, aiding in the mitigation of toxicity and the improvement of medication delivery efficacy. AI-driven methodologies provide real-time monitoring and modifications during therapy, hence enhancing patient outcomes.

From an environmental perspective, the extensive manufacture of MONPs poses several issues, especially with waste creation and resource utilization. Conventional synthesis techniques frequently employ hazardous solvents and require substantial energy consumption, resulting in carbon emissions and ecological damage. To mitigate these concerns, there is an increasing focus on sustainable production methods, including green chemistry strategies that employ cleaner, less hazardous ingredients and minimize waste. Strategies like recycling, secure disposal techniques, and the synthesis of biodegradable MONPs are being investigated to reduce the environmental impact. Implementing these sustainable procedures is essential to guarantee that the advantages of MONPs in medicinal applications do not result in environmental damage.

## Figures and Tables

**Table 1:** Recent studies on TiO_2_-based nanocarriers for cancer therapy.

S. No.	Nanocarrier	Active Drug/Therapeutic Agent	Targeted Cancer Type	Administration Method	Drug Release Within 24 h (%)	Clinical Status	References
1	TiO_2_–pPBA	Doxorubicin	Breast cancer	In vitro, In vivo	80%	Preclinical	[75]
2	TiO_2_ NPs–APTES	5-Fluorouracil	Oral cancer	In vitro	50%	Preclinical	[76]
3	PEG-coated TiO_2_ nanoparticles	Imatinib	-	In vitro	99% (pH 4.4)	Preclinical	[77]
4	PVP/PVA/TiO_2_/QC	Quercetin	Human glioblastoma	In vitro	57% (pH 5.4)	Preclinical	[78]
5	Folic acid–PEG–TiO_2_	Paclitaxel	Liver Cancer	In vitro	70%	Preclinical	[79]
6	TiO_2_–nanotubes	5-fluorouracil	Cervical cancer	In vitro	–	Preclinical	[80]
7	TiO_2_–PEG	Doxorubicin	Breast tumor	In vitro, In vivo	80%	Preclinical	[73]
8	Mesoporous TiO_2_	Doxorubicin	Lung cancer	In vitro	40%	Preclinical	[74]

**Table 2:** Recent studies on magnetic IONP-based nanocarriers for cancer therapy.

S. No.	Nanocarrier	Active Drug/Therapeutic Agent	Targeted Cancer Type	Administration Method	Drug Release Within 24 h (%)	Clinical Status	References
1	Folate–Fe_3_O_4_	Doxorubicin	Breast cancer	In vivo	-	Preclinical	[92]
2	Dextran–Fe_3_O_4_	Doxorubicin and cetuximab	lung cancer	In vivo	20%	Preclinical	[106]
3	Fe_3_O_4_ NPs with Au shell and pectin	Curcumin	Cervical cancer	In vivo	40%	Preclinical	[107]
4	Hyaluronic acid–Fe_3_O_4_	Methotrexate	Lung cancer	In vitro	8.41%	Preclinical	[108]
5	Tannic acid–Fe_3_O_4_	Doxorubicin	Colon cancer	In vitro	70%	Preclinical	[109]
6	Albumin–Fe_3_O_4_	Doxorubicin	Breast cancer	In vitro	83.35%	Preclinical	[110]
7	Fe_3_O_4_–mesoporous silica	Disulfiram	Breast cancer	In vitro	100%	Preclinical	[111]
8	Fe_3_O_4_–APTES	Methotrexate	Ovarian cancer	In vitro	95%	Preclinical	[112]
9	Cs/DOX/Cit–MNPs	Doxorubicin	Breast cancer	In vitro	75%	Preclinical	[113]
10	PMNP-VCR-FA-TF	Vincristine	Eye cancer	In vitro, ex vivo	64.71% (48 h)	Preclinical	[114]

**Table 3:** Recent studies on ZnONP-based nanocarriers for cancer therapy.

S. No.	Nanocarrier	Active Drug/Therapeutic Agent	Targeted Cancer Type	Administration Method	Drug Release Within 24 h (%)	Clinical Status	References
1	ZnONPs/DOX/FA	Doxorubicin	Colon and Breast cancer	In vitro, in vivo	-	Preclinical	[128]
2	CS/CMC/GQDs/ZnO@QC	Quercetin	Brain cancer	In vitro	82% (72 h)	Preclinical	[129]
3	5-Fu–β-CD/ZnO	5-Fluorouracil	Skin cancer	In vitro	72.5%	Preclinical	[130]
4	Pd(II)/ZnO	Palladium (II)	Breast cancer	In vitro	44.3%	Preclinical	[131]
5	PEG/PVA/ZnONPs	Quercetin	Breast cancer	In vitro	56% (pH = 5.4)	Preclinical	[118]
6	ZnO-PG-RGD	Doxorubicin	Cervical cancer	In vitro	60% (pH 5.2)	Preclinical	[132]
7	MSCNs–ZnO	Doxorubicin	Breast cancer	In vitro	35% (pH 5.0)	Preclinical	[133]
8	CH-HIS-ZnO	Quercetin	Skin cancer	In vitro	72.41% (pH 5.0)	Preclinical	[134]
9	MCC-g-PMADQUAT-g-PAcZnO	Methotrexate	Breast cancer	In vitro	32% (pH 5.4 and 40 °C)	Preclinical	[135]
10	MTX–ZnONPs	Methotrexate	Breast cancer	In vitro	90%	Preclinical	[136]

**Table 4:** Recent studies on CuONP-based nanocarriers for cancer therapy.

S. No.	Nanocarrier	Active Drug/Therapeutic Agent	Targeted Cancer Type	Administration Method	Drug Release Within 24 h (%)	Clinical Status	References
1	Ga@CuO-PTX@K-carr/FA	Paclitaxel	Breast cancer	In vitro	50% (pH 5.0)	Preclinical	[143]
2	CuO-NiO@PDA-PTX/FA NPs	Paclitaxel	Breast cancer	In vitro	40% (pH 5.0)	Preclinical	[142]
3	Zn-CuO@PTX/AlgPDA NPs	Paclitaxel	Breast cancer	In vitro	45% (pH 5.0)	Preclinical	[144]
4	CuO@BSA-MTX NPS	Methotrexate	Breast cancer	In vitro	65% (Proteinase K enzyme, pH 7.4)	Preclinical	[145]
5	CuO-PTX@PHBV-PEG	Paclitaxel	Breast cancer	In vitro	38% (pH 5.0)	Preclinical	[146]
6	PLGA-CuONPs	Doxorubicin and docetaxel	Nasopharyngeal cancer	In vitro	700 µg/ml	Preclinical	[147]
7	DOX-Cs-CuO NPs	Doxorubicin	Breast cancer	In vitro	73%	Preclinical	[148]
8	F_e3_O_4_@Cu_3_(BTC)_2_	Doxorubicin	Breast cancer	In vitro	85.5% (pH 5.0)	Preclinical	[149]

## References

[1] Brown J.S., Amend S.R., Austin R.H., Gatenby R.A., Hammarlund E.U., Pienta K.J. (2023). Updating the Definition of Cancer. Mol. Cancer Res..

[2] Soldato D., Arecco L., Agostinetto E., Franzoi M.A., Mariamidze E., Begijanashvili S., Brunetti N., Spinaci S., Solinas C., Vaz-Luis I. (2023). The Future of Breast Cancer Research in the Survivorship Field. Oncol. Ther..

[3] Deutsch M.B., Deangelis L.M., Aminoff M.J., Josephson S.A. (2014). Chapter 28—Neurologic Complications of Chemotherapy and Radiation Therapy. Aminoff’s Neurology and General Medicine.

[4] Anand U., Dey A., Chandel A.K.S., Sanyal R., Mishra A., Pandey D.K., De Falco V., Upadhyay A., Kandimalla R., Chaudhary A. (2023). Cancer Chemotherapy and beyond: Current Status, Drug Candidates, Associated Risks and Progress in Targeted Therapeutics. Genes Dis..

[5] Davodabadi F., Sajjadi S.F., Sarhadi M., Mirghasemi S., Nadali Hezaveh M., Khosravi S., Kamali Andani M., Cordani M., Basiri M., Ghavami S. (2023). Cancer Chemotherapy Resistance: Mechanisms and Recent Breakthrough in Targeted Drug Delivery. Eur. J. Pharmacol..

[6] Schirrmacher V. (2019). From Chemotherapy to Biological Therapy: A Review of Novel Concepts to Reduce the Side Effects of Systemic Cancer Treatment (Review). Int. J. Oncol..

[7] Domingues C., Santos A., Alvarez-Lorenzo C., Concheiro A., Jarak I., Veiga F., Barbosa I., Dourado M., Figueiras A. (2022). Where Is Nano Today and Where Is It Headed? A Review of Nanomedicine and the Dilemma of Nanotoxicology. ACS Nano.

[8] Sanchez-Moreno P., Ortega-Vinuesa J.L., Peula-Garcia J.M., Marchal J.A., Boulaiz H. (2018). Smart Drug-Delivery Systems for Cancer Nanotherapy. Curr. Drug Targets.

[9] Nikolova M.P., Joshi P.B., Chavali M.S. (2023). Updates on Biogenic Metallic and Metal Oxide Nanoparticles: Therapy, Drug Delivery and Cytotoxicity. Pharmaceutics.

[10] Bakhtiary Z., Saei A.A., Hajipour M.J., Raoufi M., Vermesh O., Mahmoudi M. (2016). Targeted Superparamagnetic Iron Oxide Nanoparticles for Early Detection of Cancer: Possibilities and Challenges. Nanomed. Nanotechnol. Biol. Med..

[11] Chen C., Ge J., Gao Y., Chen L., Cui J., Zeng J., Gao M. (2022). Ultrasmall Superparamagnetic Iron Oxide Nanoparticles: A next Generation Contrast Agent for Magnetic Resonance Imaging. WIREs Nanomed. Nanobiotechnol..

[12] Hu Y., Mignani S., Majoral J.-P., Shen M., Shi X. (2018). Construction of Iron Oxide Nanoparticle-Based Hybrid Platforms for Tumor Imaging and Therapy. Chem. Soc. Rev..

[13] Vats M., Mishra S.K., Baghini M.S., Chauhan D.S., Srivastava R., De A. (2017). Near Infrared Fluorescence Imaging in Nano-Therapeutics and Photo-Thermal Evaluation. Int. J. Mol. Sci..

[14] Sun L., Liu H., Ye Y., Lei Y., Islam R., Tan S., Tong R., Miao Y.-B., Cai L. (2023). Smart Nanoparticles for Cancer Therapy. Signal Transduct. Target. Ther..

[15] Choi Y.H., Han H.-K. (2018). Nanomedicines: Current Status and Future Perspectives in Aspect of Drug Delivery and Pharmacokinetics. J. Pharm. Investig..

[16] Misra K.P., Misra R.D.K. (2023). ZnO-Based Quantum Dots for Biosensing, Cancer Imaging and Therapy: An Overview. Biomed. Mater. Devices.

[17] Khan M.J., Ahmad A., Khan M.A., Siddiqui S. (2021). Zinc Oxide Nanoparticle Induces Apoptosis in Human Epidermoid Carcinoma Cells Through Reactive Oxygen Species and DNA Degradation. Biol. Trace Elem. Res..

[18] Patel S., Sahai S.K., Hagberg C., Gottumukkala V., Riedel B., Nates J., Buggy D. (2023). 4—Traditional Cancer Therapies and Perioperative Implications. Perioperative Care of the Cancer Patient.

[19] Ullah M., Shah M.I., Hasan M.W., Jamshed M., Mustafa U., Inam M. (2024). Engineered Metal Nanoparticles for Precision Drug Delivery: Pioneering the Future of Medicine: Mini Review. J. Chin. Chem. Soc..

[20] Erduran M., Çankaya N., Yalcin S., Ahmed S. (2024). Biobased Nanomaterials in Drug Delivery. Biobased Nanomaterials: Applications in Biomedicine, Food Industry, Agriculture, and Environmental Sustainability.

[21] Hosseinpour-Moghadam R., Taghizadeh F., Goshtasbi N., Merati F., Haeri A., Barabadi H., Mostafavi E., Mustansar Hussain C. (2024). Chapter 23—Applications of Liposomes for Overcoming Cancer Drug Resistance. Functionalized Nanomaterials for Cancer Research.

[22] Rajkumar S., Prabaharan M. (2018). Multi-Functional Nanocarriers Based on Iron Oxide Nanoparticles Conjugated with Doxorubicin, Poly(Ethylene Glycol) and Folic Acid as Theranostics for Cancer Therapy. Colloids Surf. B Biointerfaces.

[23] Predoi D., Balas M., Badea M.A., Ciobanu S.C., Buton N., Dinischiotu A. (2023). Dextran-Coated Iron Oxide Nanoparticles Loaded with 5-Fluorouracil for Drug-Delivery Applications. Nanomaterials.

[24] Chen J., Wu Q., Luo L., Wang Y., Zhong Y., Dai H.-B., Sun D., Luo M.-L., Wu W., Wang G.-X. (2017). Dual Tumor-Targeted Poly(Lactic-Co-Glycolic Acid)–Polyethylene Glycol–Folic Acid Nanoparticles: A Novel Biodegradable Nanocarrier for Secure and Efficient Antitumor Drug Delivery. Int. J. Nanomed..

[25] Park J., Kadasala N.R., Abouelmagd S.A., Castanares M.A., Collins D.S., Wei A., Yeo Y. (2016). Polymer–Iron Oxide Composite Nanoparticles for EPR-Independent Drug Delivery. Biomaterials.

[26] Xie J., Zhang Y., Yan C., Song L., Wen S., Zang F., Chen G., Ding Q., Yan C., Gu N. (2014). High-Performance PEGylated Mn–Zn Ferrite Nanocrystals as a Passive-Targeted Agent for Magnetically Induced Cancer Theranostics. Biomaterials.

[27] Mu Q., Lin G., Patton V.K., Wang K., Press O.W., Zhang M. (2016). Gemcitabine and Chlorotoxin Conjugated Iron Oxide Nanoparticles for Glioblastoma Therapy. J. Mater. Chem. B.

[28] Razavi M.S., Abdollahi A., Malek-Khatabi A., Ejarestaghi N.M., Atashi A., Yousefi N., Ebrahimnejad P., Elsawy M.A., Dinarvand R. (2023). Recent Advances in PLGA-Based Nanofibers as Anticancer Drug Delivery Systems. J. Drug Deliv. Sci. Technol..

[29] Dang B.-T.N., Kwon T.K., Lee S., Jeong J.-H., Yook S. (2024). Nanoparticle-Based Immunoengineering Strategies for Enhancing Cancer Immunotherapy. J. Control. Release.

[30] Wang J., Li Y., Nie G. (2021). Multifunctional Biomolecule Nanostructures for Cancer Therapy. Nat. Rev. Mater..

[31] Maghsoudnia N., Eftekhari R.B., Sohi A.N., Zamzami A., Dorkoosh F.A. (2020). Application of Nano-Based Systems for Drug Delivery and Targeting: A Review. J. Nanoparticle Res..

[32] Sundar D.S., Antoniraj M.G., Kumar C.S., Mohapatra S.S., Houreld N.N., Ruckmani K. (2016). Recent Trends of Biocompatible and Biodegradable Nanoparticles in Drug Delivery: A Review. Curr. Med. Chem..

[33] Ezike T.C., Okpala U.S., Onoja U.L., Nwike C.P., Ezeako E.C., Okpara O.J., Okoroafor C.C., Eze S.C., Kalu O.L., Odoh E.C. (2023). Advances in Drug Delivery Systems, Challenges and Future Directions. Heliyon.

[34] Yurtdaş Kırımlıoğlu G., Kesharwani P. (2023). Chapter 3—Drug Loading Methods and Drug Release Mechanisms of PLGA Nanoparticles. Poly(Lactic-Co-Glycolic Acid) (PLGA) Nanoparticles for Drug Delivery.

[35] Thang N.H., Chien T.B., Cuong D.X. (2023). Polymer-Based Hydrogels Applied in Drug Delivery: An Overview. Gels.

[36] Rizwanullah M., Ahmad M.Z., Ghoneim M.M., Alshehri S., Imam S.S., Md S., Alhakamy N.A., Jain K., Ahmad J. (2021). Receptor-Mediated Targeted Delivery of Surface-ModifiedNanomedicine in Breast Cancer: Recent Update and Challenges. Pharmaceutics.

[37] Tian Y., Wang X., Wu C., Qiao J., Jin H., Li H. (2024). A Protracted War against Cancer Drug Resistance. Cancer Cell Int..

[38] Yu S., Zheng J., Zhang Y., Meng D., Wang Y., Xu X., Liang N., Shabiti S., Zhang X., Wang Z. (2023). The Mechanisms of Multidrug Resistance of Breast Cancer and Research Progress on Related Reversal Agents. Bioorg. Med. Chem..

[39] Patel D., Sethi N., Patel P., Shah S., Patel K. (2024). Exploring the Potential of P-Glycoprotein Inhibitors in the Targeted Delivery of Anti-Cancer Drugs: A Comprehensive Review. Eur. J. Pharm. Biopharm..

[40] Ashique S., Garg A., Hussain A., Farid A., Kumar P., Taghizadeh-Hesary F. (2023). Nanodelivery Systems: An Efficient and Target-Specific Approach for Drug-Resistant Cancers. Cancer Med..

[41] Nyamba I., Sombié C.B., Yabré M., Zimé-Diawara H., Yaméogo J., Ouédraogo S., Lechanteur A., Semdé R., Evrard B. (2024). Pharmaceutical Approaches for Enhancing Solubility and Oral Bioavailability of Poorly Soluble Drugs. Eur. J. Pharm. Biopharm..

[42] Bhalani D.V., Nutan B., Kumar A., Singh Chandel A.K. (2022). Bioavailability Enhancement Techniques for Poorly Aqueous Soluble Drugs and Therapeutics. Biomedicines.

[43] Sood R., Tomar D., Kaushik P., Sharma P., Rani N., Guarve K., Dhankhar S., Garg N. (2024). Enhanced Solubility and Increased Bioavailability with Engineered Nanocrystals. Curr. Drug Ther..

[44] Jayapriya P., Pardhi E., Vasave R., Guru S.K., Madan J., Mehra N.K. (2023). A Review on Stimuli-pH Responsive Liposomal Formulation in Cancer Therapy. J. Drug Deliv. Sci. Technol..

[45] Hari S.K., Gauba A., Shrivastava N., Tripathi R.M., Jain S.K., Pandey A.K. (2023). Polymeric Micelles and Cancer Therapy: An Ingenious Multimodal Tumor-Targeted Drug Delivery System. Drug Deliv. Transl. Res..

[46] Marabada D., Li J., Wei S., Huang Q., Wang Z. (2023). Cyclodextrin Based Nanoparticles for Smart Drug Delivery in Colorectal Cancer. Chem. Biol. Drug Des..

[47] Virmani T., Kumar G., Sharma A., Pathak K., Akhtar M.S., Afzal O., Altamimi A.S.A. (2023). Amelioration of Cancer Employing Chitosan, Its Derivatives, and Chitosan-Based Nanoparticles: Recent Updates. Polymers.

[48] Safitri N., Rauf N., Tahir D. (2023). Enhancing Drug Loading and Release with Hydroxyapatite Nanoparticles for Efficient Drug Delivery: A Review Synthesis Methods, Surface Ion Effects, and Clinical Prospects. J. Drug Deliv. Sci. Technol..

[49] Duncan R., Richardson S.C.W. (2012). Endocytosis and Intracellular Trafficking as Gateways for Nanomedicine Delivery: Opportunities and Challenges. Mol. Pharm..

[50] Gavas S., Quazi S., Karpiński T.M. (2021). Nanoparticles for Cancer Therapy: Current Progress and Challenges. Nanoscale Res. Lett..

[51] Triphati A., Pirzadah T.B., Ozturk M., Roy A., Bhat R.A., Vardar-Sukan F., Policarpo Tonelli F.M. (2023). Chapter 3—Synthesis Methods of Nanoparticles and Their Key Applications. Synthesis of Bionanomaterials for Biomedical Applications.

[52] El-Khawaga A.M., Zidan A., El-Mageed A.I.A.A. (2023). Preparation Methods of Different Nanomaterials for Various Potential Applications: A Review. J. Mol. Struct..

[53] Shreyash N., Sonker M., Bajpai S., Tiwary S.K. (2021). Review of the Mechanism of Nanocarriers and Technological Developments in the Field of Nanoparticles for Applications in Cancer Theragnostics. ACS Appl. Bio Mater..

[54] Upadhyay K., Tamrakar R.K., Thomas S., Kumar M. (2023). Surface Functionalized Nanoparticles: A Boon to Biomedical Science. Chem. Biol. Interact..

[55] Aminzai M.T., Yildirim M., Yabalak E. (2024). Metallic Nanoparticles Unveiled: Synthesis, Characterization, and Their Environmental, Medicinal, and Agricultural Applications. Talanta.

[56] Roberts E.J., Karadaghi L.R., Wang L., Malmstadt N., Brutchey R.L. (2019). Continuous Flow Methods of Fabricating Catalytically Active Metal Nanoparticles. ACS Appl. Mater. Interfaces.

[57] Niculescu A.-G., Chircov C., Grumezescu A.M. (2022). Magnetite Nanoparticles: Synthesis Methods—A Comparative Review. Methods.

[58] Naik R., Nagaswarupa H.P., Darukesha B.H.M., Tejashwini D.M., Naik R., Nagaswarupa H.P., Darukesha B.H.M., Tejashwini D.M. (2024). Fundamentals of Nanomaterial Synthesis. Advances in Space Radiation Detection: Novel Nanomaterials and Techniques.

[59] Liu H., Wang S., Yang J., Zhuo R., Zhao J., Liu L., Li Y. (2024). The Application of Supercritical Fluid Technology in the Synthesis of Metal and Metal Oxide Nanoparticles. CrystEngComm.

[60] Zhang X., Ng H.L.H., Lu A., Lin C., Zhou L., Lin G., Zhang Y., Yang Z., Zhang H. (2016). Drug Delivery System Targeting Advanced Hepatocellular Carcinoma: Current and Future. Nanomed. Nanotechnol. Biol. Med..

[61] Hejmady S., Pradhan R., Alexander A., Agrawal M., Singhvi G., Gorain B., Tiwari S., Kesharwani P., Dubey S.K. (2020). Recent Advances in Targeted Nanomedicine as Promising Antitumor Therapeutics. Drug Discov. Today.

[62] Chen S., Cao R., Xiang L., Li Z., Chen H., Zhang J., Feng X. (2023). Research Progress in Nucleus-Targeted Tumor Therapy. Biomater. Sci..

[63] Torchilin V.P., Schäfer-Korting M. (2010). Passive and Active Drug Targeting: Drug Delivery to Tumors as an Example. Drug Delivery.

[64] Yadav K.S., Mishra D.K., Deshpande A., Pethe A.M., Tekade R.K. (2019). Chapter 7—Levels of Drug Targeting. Basic Fundamentals of Drug Delivery.

[65] Banushi B., Joseph S.R., Lum B., Lee J.J., Simpson F. (2023). Endocytosis in Cancer and Cancer Therapy. Nat. Rev. Cancer.

[66] Dhivya R., Ranjani J., Bowen P.K., Rajendhran J., Mayandi J., Annaraj J. (2017). Biocompatible Curcumin Loaded PMMA-PEG/ZnO Nanocomposite Induce Apoptosis and Cytotoxicity in Human Gastric Cancer Cells. Mater. Sci. Eng. C.

[67] Sathiyaseelan A., Saravanakumar K., Wang M.-H. (2022). Cerium Oxide Decorated 5-Fluorouracil Loaded Chitosan Nanoparticles for Treatment of Hepatocellular Carcinoma. Int. J. Biol. Macromol..

[68] Liu M., Sun X., Liao Z., Li Y., Qi X., Qian Y., Fenniri H., Zhao P., Shen J. (2019). Zinc Oxide End-Capped Fe_3_O_4_@mSiO_2_ Core-Shell Nanocarriers as Targeted and Responsive Drug Delivery System for Chemo-/Ions Synergistic Therapeutics. Drug Deliv..

[69] Bertrand N., Leroux J.-C. (2012). The Journey of a Drug-Carrier in the Body: An Anatomo-Physiological Perspective. J. Control. Release.

[70] Salahpour Anarjan F. (2019). Active Targeting Drug Delivery Nanocarriers: Ligands. Nano-Struct. Nano-Objects.

[71] Raja G., Cao S., Kim D.-H., Kim T.-J. (2020). Mechanoregulation of Titanium Dioxide Nanoparticles in Cancer Therapy. Mater. Sci. Eng. C.

[72] Tonelli F.M.P., Tonelli F.C.P., Cordeiro H.G. (2024). TiO_2_ Nanoparticles in Cancer Therapy as Nanocarriers in Paclitaxel’s Delivery and Nanosensitizers in Phototherapies and/or Sonodynamic Therapy. Curr. Pharm. Biotechnol..

[73] Du Y., Ren W., Li Y., Zhang Q., Zeng L., Chi C., Wu A., Tian J. (2015). The Enhanced Chemotherapeutic Effects of Doxorubicin Loaded PEG Coated TiO_2_ Nanocarriers in an Orthotopic Breast Tumor Bearing Mouse Model. J. Mater. Chem. B.

[74] Guo Z., Zheng K., Tan Z., Liu Y., Zhao Z., Zhu G., Ma K., Cui C., Wang L., Kang T. (2018). Overcoming Drug Resistance with Functional Mesoporous Titanium Dioxide Nanoparticles Combining Targeting, Drug Delivery and Photodynamic Therapy. J. Mater. Chem. B.

[75] Kim S., Im S., Park E.-Y., Lee J., Kim C., Kim T., Kim W.J. (2020). Drug-Loaded Titanium Dioxide Nanoparticle Coated with Tumor Targeting Polymer as a Sonodynamic Chemotherapeutic Agent for Anti-Cancer Therapy. Nanomed. Nanotechnol. Biol. Med..

[76] Rivera Rodriguez M.A., Campos-Ibarra V., Rasu Chettiar A.-D., Marasamy L., Manisekaran R. (2024). Anticancer Effect of Surface Functionalized Nano Titanium Dioxide with 5-Fluorouracil on Oral Cancer Cell Line—An in Vitro Study. Micro Nano Lett..

[77] Bhullar S., Goyal N., Gupta S. (2024). FericipXT-Coated PEGylated Rutile TiO_2_ Nanoparticles in Drug Delivery: In Vitro Assessment of Imatinib Release. RSC Adv..

[78] Eshaghi M.M., Pourmadadi M., Rahdar A., Díez-Pascual A.M. (2023). Improving Quercetin Anticancer Activity through a Novel Polyvinylpyrrolidone/Polyvinyl Alcohol/TiO_2_ Nanocomposite. J. Drug Deliv. Sci. Technol..

[79] Devanand Venkatasubbu G., Ramasamy S., Ramakrishnan V., Kumar J. (2013). Folate Targeted PEGylated Titanium Dioxide Nanoparticles as a Nanocarrier for Targeted Paclitaxel Drug Delivery. Adv. Powder Technol..

[80] Heidari Khoee M., Khoee S., Lotfi M. (2019). Synthesis of Titanium Dioxide Nanotubes with Liposomal Covers for Carrying and Extended Release of 5-FU as Anticancer Drug in the Treatment of HeLa Cells. Anal. Biochem..

[81] Kenchegowda M., Rahamathulla M., Hani U., Begum M.Y., Guruswamy S., Osmani R.A.M., Gowrav M.P., Alshehri S., Ghoneim M.M., Alshlowi A. (2022). Smart Nanocarriers as an Emerging Platform for Cancer Therapy: A Review. Molecules.

[82] Senapati S., Mahanta A.K., Kumar S., Maiti P. (2018). Controlled Drug Delivery Vehicles for Cancer Treatment and Their Performance. Signal Transduct. Target. Ther..

[83] Chandrakala V., Aruna V., Angajala G. (2022). Review on Metal Nanoparticles as Nanocarriers: Current Challenges and Perspectives in Drug Delivery Systems. Emergent Mater..

[84] Liang P.-C., Chen Y.-C., Chiang C.-F., Mo L.-R., Wei S.-Y., Hsieh W.-Y., Lin W.-L. (2016). Doxorubicin-Modified Magnetic Nanoparticles as a Drug Delivery System for Magnetic Resonance Imaging-Monitoring Magnet-Enhancing Tumor Chemotherapy. Int. J. Nanomed..

[85] El Ghany Y.I.A., Tawfik M.M., El Bous M., Gomaa I., Moustafa A.M.Y., Hosny N.M., El-Dossoki F., Hassan M., Shehata A. (2024). Sesuvium sesuvioides (Fenzl) Mediated Synthesis of Zinc Oxide and Copper Oxide Nanoparticles and Their Potential Cytotoxic and Apoptotic Effects. The First International Conference & Expo on Green Sciences.

[86] Truong T.T., Mondal S., Doan V.H.M., Tak S., Choi J., Oh H., Nguyen T.D., Misra M., Lee B., Oh J. (2024). Precision-Engineered Metal and Metal-Oxide Nanoparticles for Biomedical Imaging and Healthcare Applications. Adv. Colloid Interface Sci..

[87] Mi Y., Liu X., Zhao J., Ding J., Feng S.-S. (2012). Multimodality Treatment of Cancer with Herceptin Conjugated, Thermomagnetic Iron Oxides and Docetaxel Loaded Nanoparticles of Biodegradable Polymers. Biomaterials.

[88] Liu G., Gao J., Ai H., Chen X. (2013). Applications and Potential Toxicity of Magnetic Iron Oxide Nanoparticles. Small.

[89] Saeed M., Ren W., Wu A. (2018). Therapeutic Applications of Iron Oxide Based Nanoparticles in Cancer: Basic Concepts and Recent Advances. Biomater. Sci..

[90] Su C. (2017). Environmental Implications and Applications of Engineered Nanoscale Magnetite and Its Hybrid Nanocomposites: A Review of Recent Literature. J. Hazard. Mater..

[91] Montiel Schneider M.G., Martín M.J., Otarola J., Vakarelska E., Simeonov V., Lassalle V., Nedyalkova M. (2022). Biomedical Applications of Iron Oxide Nanoparticles: Current Insights Progress and Perspectives. Pharmaceutics.

[92] Pourradi N.M.A., Babaei H., Hamishehkar H., Baradaran B., Shokouhi-Gogani B., Shanehbandi D., Ghorbani M., Azarmi Y. (2022). Targeted Delivery of Doxorubicin by Thermo/pH-Responsive Magnetic Nanoparticles in a Rat Model of Breast Cancer. Toxicol. Appl. Pharmacol..

[93] Rarokar N., Yadav S., Saoji S., Bramhe P., Agade R., Gurav S., Khedekar P., Subramaniyan V., Wong L.S., Kumarasamy V. (2024). Magnetic Nanosystem a Tool for Targeted Delivery and Diagnostic Application: Current Challenges and Recent Advancement. Int. J. Pharm. X.

[94] Wagstaff A.J., Brown S.D., Holden M.R., Craig G.E., Plumb J.A., Brown R.E., Schreiter N., Chrzanowski W., Wheate N.J. (2012). Cisplatin Drug Delivery Using Gold-Coated Iron Oxide Nanoparticles for Enhanced Tumour Targeting with External Magnetic Fields. Inorg. Chim. Acta.

[95] Nadeem M., Ahmad M., Akhtar M.S., Shaari A., Riaz S., Naseem S., Masood M., Saeed M.A. (2016). Magnetic Properties of Polyvinyl Alcohol and Doxorubicine Loaded Iron Oxide Nanoparticles for Anticancer Drug Delivery Applications. PLoS ONE.

[96] Yetisgin A.A., Cetinel S., Zuvin M., Kosar A., Kutlu O. (2020). Therapeutic Nanoparticles and Their Targeted Delivery Applications. Molecules.

[97] Kara N., Ayoub N., Ilgu H., Fotiadis D., Ilgu M. (2023). Aptamers Targeting Membrane Proteins for Sensor and Diagnostic Applications. Molecules.

[98] Nagesh P.K.B., Johnson N.R., Boya V.K.N., Chowdhury P., Othman S.F., Khalilzad-Sharghi V., Hafeez B.B., Ganju A., Khan S., Behrman S.W. (2016). PSMA Targeted Docetaxel-Loaded Superparamagnetic Iron Oxide Nanoparticles for Prostate Cancer. Colloids Surf. B Biointerfaces.

[99] Aires A., Ocampo S.M., Simões B.M., Rodríguez M.J., Cadenas J.F., Couleaud P., Spence K., Latorre A., Miranda R., Somoza Á. (2016). Multifunctionalized Iron Oxide Nanoparticles for Selective Drug Delivery to CD44-Positive Cancer Cells. Nanotechnology.

[100] Huang Y., Mao K., Zhang B., Zhao Y. (2017). Superparamagnetic Iron Oxide Nanoparticles Conjugated with Folic Acid for Dual Target-Specific Drug Delivery and MRI in Cancer Theranostics. Mater. Sci. Eng. C.

[101] Yang Y., Guo Q., Peng J., Su J., Lu X., Zhao Y., Qian Z. (2016). Doxorubicin-Conjugated Heparin-Coated Superparamagnetic Iron Oxide Nanoparticles for Combined Anticancer Drug Delivery and Magnetic Resonance Imaging. J. Biomed. Nanotechnol..

[102] Abri Aghdam M., Bagheri R., Mosafer J., Baradaran B., Hashemzaei M., Baghbanzadeh A., de la Guardia M., Mokhtarzadeh A. (2019). Recent Advances on Thermosensitive and pH-Sensitive Liposomes Employed in Controlled Release. J. Control. Release.

[103] Munnier E., Cohen-Jonathan S., Linassier C., Douziech-Eyrolles L., Marchais H., Soucé M., Hervé K., Dubois P., Chourpa I. (2008). Novel Method of Doxorubicin–SPION Reversible Association for Magnetic Drug Targeting. Int. J. Pharm..

[104] Zou Y., Liu P., Liu C.-H., Zhi X.-T. (2015). Doxorubicin-Loaded Mesoporous Magnetic Nanoparticles to Induce Apoptosis in Breast Cancer Cells. Biomed. Pharmacother..

[105] Quinto C.A., Mohindra P., Tong S., Bao G. (2015). Multifunctional Superparamagnetic Iron Oxide Nanoparticles for Combined Chemotherapy and Hyperthermia Cancer Treatment. Nanoscale.

[106] Zhang Q., Liu Q., Du M., Vermorken A., Cui Y., Zhang L., Guo L., Ma L., Chen M. (2019). Cetuximab and Doxorubicin Loaded Dextran-Coated Fe3O4 Magnetic Nanoparticles as Novel Targeted Nanocarriers for Non-Small Cell Lung Cancer. J. Magn. Magn. Mater..

[107] Sood A., Arora V., Kumari S., Sarkar A., Kumaran S.S., Chaturvedi S., Jain T.K., Agrawal G. (2021). Imaging Application and Radiosensitivity Enhancement of Pectin Decorated Multifunctional Magnetic Nanoparticles in Cancer Therapy. Int. J. Biol. Macromol..

[108] Dou J., Mi Y., Daneshmand S., Heidari Majd M. (2022). The Effect of Magnetic Nanoparticles Containing Hyaluronic Acid and Methotrexate on the Expression of Genes Involved in Apoptosis and Metastasis in A549 Lung Cancer Cell Lines. Arab. J. Chem..

[109] Gonbadi P., Jalal R., Akhlaghinia B., Ghasemzadeh M.S. (2022). Tannic Acid-Modified Magnetic Hydrotalcite-Based MgAl Nanoparticles for the in Vitro Targeted Delivery of Doxorubicin to the Estrogen Receptor-Overexpressing Colorectal Cancer Cells. J. Drug Deliv. Sci. Technol..

[110] Hormozi N., Esmaeili A. (2019). Synthesis and Correction of Albumin Magnetic Nanoparticles with Organic Compounds for Absorbing and Releasing Doxorubicin Hydrochloride. Colloids Surf. B Biointerfaces.

[111] Solak K., Mavi A., Yılmaz B. (2021). Disulfiram-Loaded Functionalized Magnetic Nanoparticles Combined with Copper and Sodium Nitroprusside in Breast Cancer Cells. Mater. Sci. Eng. C.

[112] Nowak-Jary J., Płóciennik A., Machnicka B. (2024). Functionalized Magnetic Fe3O4 Nanoparticles for Targeted Methotrexate Delivery in Ovarian Cancer Therapy. Int. J. Mol. Sci..

[113] Akl M.A., Kamel A.M., El-Ghaffar M.A.A. (2023). Biodegradable Functionalized Magnetite Nanoparticles as Binary-Targeting Carrier for Breast Carcinoma. BMC Chem..

[114] Sadri E., Khoee S., Moayeri S., Haji Ali B., Pirhajati Mahabadi V., Shirvalilou S., Khoei S. (2023). Enhanced Anti-Tumor Activity of Transferrin/Folate Dual-Targeting Magnetic Nanoparticles Using Chemo-Thermo Therapy on Retinoblastoma Cancer Cells Y79. Sci. Rep..

[115] Anjum S., Hashim M., Malik S.A., Khan M., Lorenzo J.M., Abbasi B.H., Hano C. (2021). Recent Advances in Zinc Oxide Nanoparticles (ZnO NPs) for Cancer Diagnosis, Target Drug Delivery, and Treatment. Cancers.

[116] Gupta J., Hassan P.A., Barick K.C. (2023). Multifunctional ZnO Nanostructures: A next Generation Nanomedicine for Cancer Therapy, Targeted Drug Delivery, Bioimaging, and Tissue Regeneration. Nanotechnology.

[117] Singh T.A., Das J., Sil P.C. (2020). Zinc Oxide Nanoparticles: A Comprehensive Review on Its Synthesis, Anticancer and Drug Delivery Applications as Well as Health Risks. Adv. Colloid Interface Sci..

[118] Gholami A., Pourmadadi. M., Abdouss. H., Amiri. Z., Abdouss. M., Rahdar A., Behzadmehr. R., Pandey S. (2024). Formulation of Double Microemulsion Based on pH-Responsive PEG/PVA/Zinc Oxide as a Potential Nano-Platform for Drug Delivery: Green Synthesis, and Physico-Chemical Characterization. J. Mol. Liq..

[119] Zheng C., Wang Y., Phua S.Z.F., Lim W.Q., Zhao Y. (2017). ZnO–DOX@ZIF-8 Core–Shell Nanoparticles for pH-Responsive Drug Delivery. ACS Biomater. Sci. Eng..

[120] Cai X., Luo Y., Zhang W., Du D., Lin Y. (2016). pH-Sensitive ZnO Quantum Dots–Doxorubicin Nanoparticles for Lung Cancer Targeted Drug Delivery. ACS Appl. Mater. Interfaces.

[121] Tan L., Liu J., Zhou W., Wei J., Peng Z. (2014). A Novel Thermal and pH Responsive Drug Delivery System Based on ZnO@PNIPAM Hybrid Nanoparticles. Mater. Sci. Eng. C.

[122] Mishra P., Ali Ahmad M.F., Al-Keridis L.A., Saeed M., Alshammari N., Alabdallah N.M., Tiwari R.K., Ahmad A., Verma M., Fatima S. (2023). Methotrexate-Conjugated Zinc Oxide Nanoparticles Exert a Substantially Improved Cytotoxic Effect on Lung Cancer Cells by Inducing Apoptosis. Front. Pharmacol..

[123] Guo D., Wu C., Jiang H., Li Q., Wang X., Chen B. (2008). Synergistic Cytotoxic Effect of Different Sized ZnO Nanoparticles and Daunorubicin against Leukemia Cancer Cells under UV Irradiation. J. Photochem. Photobiol. B.

[124] Al Dine E.J., Marchal S., Schneider R., Hamie B., Ghanbaja J., Roques-Carmes T., Hamieh T., Toufaily J., Gaffet E., Alem H. (2018). A Facile Approach for Doxorubicine Delivery in Cancer Cells by Responsive and Fluorescent Core/Shell Quantum Dots. Bioconjugate Chem..

[125] Li C., Zhang H., Gong X., Li Q., Zhao X. (2019). Synthesis, Characterization, and Cytotoxicity Assessment of N-Acetyl-l-Cysteine Capped ZnO Nanoparticles as Camptothecin Delivery System. Colloids Surf. B Biointerfaces.

[126] Peng H., Cui B., Li G., Wang Y., Li N., Chang Z., Wang Y. (2015). A Multifunctional β-CD-Modified Fe_3_O_4_@ZnO:Er^3+^,Yb^3+^ Nanocarrier for Antitumor Drug Delivery and Microwave-Triggered Drug Release. Mater. Sci. Eng. C.

[127] Zhao W., Wei J.-S., Zhang P., Chen J., Kong J.-L., Sun L.-H., Xiong H.-M., Möhwald H. (2017). Self-Assembled ZnO Nanoparticle Capsules for Carrying and Delivering Isotretinoin to Cancer Cells. ACS Appl. Mater. Interfaces.

[128] Gomaa S., Nassef M., Tabl G., Zaki S., Abdel-Ghany A. (2024). Doxorubicin and Folic Acid-Loaded Zinc Oxide Nanoparticles-Based Combined Anti-Tumor and Anti-Inflammatory Approach for Enhanced Anti-Cancer Therapy. BMC Cancer.

[129] Ostovar S., Pourmadadi M., Zaker M.A. (2023). Co-Biopolymer of Chitosan/Carboxymethyl Cellulose Hydrogel Improved by Zinc Oxide and Graphene Quantum Dots Nanoparticles as pH-Sensitive Nanocomposite for Quercetin Delivery to Brain Cancer Treatment. Int. J. Biol. Macromol..

[130] Kaliyaperumal V., Rajasekaran S., Kanniah R., Gopal D., Ayyakannu Sundaram G., Kumar A.S.K. (2024). Synthesis and Evaluation of Gelatin–Chitosan Biofilms Incorporating Zinc Oxide Nanoparticles and 5-Fluorouracil for Cancer Treatment. Materials.

[131] Mosleh A.M., El-Sherif A.A., El-Sayed A.A., Fahmy H.M. (2024). Characterization and Cytotoxicity Assessment of Synthesized Palladium (II) Complex-Encapsulated Zinc Oxide Nanoparticles for Cancer Treatment. Cell Biochem. Biophys..

[132] Yang X., Zhang C., Li A., Wang J., Cai X. (2019). Red Fluorescent ZnO Nanoparticle Grafted with Polyglycerol and Conjugated RGD Peptide as Drug Delivery Vehicles for Efficient Target Cancer Therapy. Mater. Sci. Eng. C.

[133] Chen M., Hu J., Bian C., Zhu C., Chen C., Guo Z., Zhang Z., Agyekum G.A., Zhang Z., Cao X. (2020). pH-Responsive and Biodegradable ZnO-Capped Mesoporous Silica Composite Nanoparticles for Drug Delivery. Materials.

[134] George D., Maheswari P.U., Begum K.M.M.S. (2020). Chitosan-Cellulose Hydrogel Conjugated with L-Histidine and Zinc Oxide Nanoparticles for Sustained Drug Delivery: Kinetics and In-Vitro Biological Studies. Carbohydr. Polym..

[135] Abbasian M., Hasanzadeh P., Mahmoodzadeh F., Salehi R. (2020). Novel Cationic Cellulose-Based Nanocomposites for Targeted Delivery of Methotrexate to Breast Cancer Cells. J. Macromol. Sci. Part A.

[136] Joshi M., Bhatt P. (2023). Deciphering the Anticancer Activity of Biocompatible Zinc Oxide Nanoparticles Loaded with Methotrexate on Breast Cancer Cells. Bull. Mater. Sci..

[137] Gebreslassie Y.T., Gebremeskel F.G. (2024). Green and Cost-Effective Biofabrication of Copper Oxide Nanoparticles: Exploring Antimicrobial and Anticancer Applications. Biotechnol. Rep..

[138] Zaiki Y., Iskandar A., Wong T.W. (2023). Functionalized Chitosan for Cancer Nano Drug Delivery. Biotechnol. Adv..

[139] Atloo T., Mohammadkhani R., Mohammadi A., Zaboli K.A., Kaboli S., Rahimi H., Nosrati H., Danafar H. (2022). The Bovine Serum Albumin Coated Copper Oxide Nanoparticle for Curcumin Delivery in Biological Environment: In-Vitro Drug Release. J. Polym. Environ..

[140] Mariadoss A.V.A., Saravanakumar K., Sathiyaseelan A., Venkatachalam K., Wang M.-H. (2020). Folic Acid Functionalized Starch Encapsulated Green Synthesized Copper Oxide Nanoparticles for Targeted Drug Delivery in Breast Cancer Therapy. Int. J. Biol. Macromol..

[141] Goswami U., Dutta A., Raza A., Kandimalla R., Kalita S., Ghosh S.S., Chattopadhyay A. (2018). Transferrin–Copper Nanocluster–Doxorubicin Nanoparticles as Targeted Theranostic Cancer Nanodrug. ACS Appl. Mater. Interfaces.

[142] Singh S., Pal K. (2023). Exploration of Polydopamine Capped Bimetallic Oxide (CuO-NiO) Nanoparticles Inspired by Mussels for Enhanced and Targeted Paclitaxel Delivery for Synergistic Breast Cancer Therapy. Appl. Surf. Sci..

[143] Singh S., Pal K. (2024). Polyphenol Modified CuO Nanorods Capped by Kappa-Carrageenan for Controlled Paclitaxel Release in Furnishing Targeted Chemotherapy in Breast Carcinoma Cells. Int. J. Biol. Macromol..

[144] Singh S., Pal K. (2023). Folic-Acid Adorned Alginate-Polydopamine Modified Paclitaxel/Zn-CuO Nanocomplex for pH Triggered Drug Release and Synergistic Antitumor Efficacy. Int. J. Biol. Macromol..

[145] Mohammadhassan Z., Mohammadkhani R., Mohammadi A., Zaboli K.A., Kaboli S., Rahimi H., Nosrati H., Danafar H. (2022). Preparation of Copper Oxide Nanoparticles Coated with Bovine Serum Albumin for Delivery of Methotrexate. J. Drug Deliv. Sci. Technol..

[146] Singh S., Ghosh C., Roy P., Pal K. (2022). Biosynthesis of Folic Acid Appended PHBV Modified Copper Oxide Nanorods for pH Sensitive Drug Release in Targeted Breast Cancer Therapy. Int. J. Pharm..

[147] Guo L.-M., Xu X.-M., Zhao D., Cai X.-G., Zhou B. (2020). Biosynthesis, Characterization of PLGA Coated Folate-Mediated Multiple Drug Loaded Copper Oxide (CuO) Nanoparticles and It’s Cytotoxicity on Nasopharyngeal Cancer Cell Lines. AMB Express.

[148] Varukattu N.B., Vivek R., Rejeeth C., Thangam R., Ponraj T., Sharma A., Kannan S. (2020). Nanostructured pH-Responsive Biocompatible Chitosan Coated Copper Oxide Nanoparticles: A Polymeric Smart Intracellular Delivery System for Doxorubicin in Breast Cancer Cells. Arab. J. Chem..

[149] Gharehdaghi Z., Rahimi R., Naghib S.M., Molaabasi F. (2022). Fabrication and Application of Copper Metal–Organic Frameworks as Nanocarriers for pH-Responsive Anticancer Drug Delivery. J. Iran. Chem. Soc..

[150] Peng N., Ding X., Wang Z., Cheng Y., Gong Z., Xu X., Gao X., Cai Q., Huang S., Liu Y. (2019). Novel Dual Responsive Alginate-Based Magnetic Nanogels for Onco-Theranostics. Carbohydr. Polym..

[151] Zhang Y., Guo C., Liu L., Xu J., Jiang H., Li D., Lan J., Li J., Yang J., Tu Q. (2020). ZnO-Based Multifunctional Nanocomposites to Inhibit Progression and Metastasis of Melanoma by Eliciting Antitumor Immunity via Immunogenic Cell Death. Theranostics.

[152] Liu Z., Wan P., Yang M., Han F., Wang T., Wang Y., Li Y. (2021). Cell Membrane Camouflaged Cerium Oxide Nanocubes for Targeting Enhanced Tumor-Selective Therapy. J. Mater. Chem. B.

[153] Alshawwa S.Z., Kassem A.A., Farid R.M., Mostafa S.K., Labib G.S. (2022). Nanocarrier Drug Delivery Systems: Characterization, Limitations, Future Perspectives and Implementation of Artificial Intelligence. Pharmaceutics.

[154] Kommineni N., Chaudhari R., Conde J., Tamburaci S., Cecen B., Chandra P., Prasad R. (2023). Engineered Liposomes in Interventional Theranostics of Solid Tumors. ACS Biomater. Sci. Eng..

[155] Fernandes C., Suares D., Yergeri M.C. (2018). Tumor Microenvironment Targeted Nanotherapy. Front. Pharmacol..

[156] Elumalai K., Srinivasan S., Shanmugam A. (2024). Review of the Efficacy of Nanoparticle-Based Drug Delivery Systems for Cancer Treatment. Biomed. Technol..

[157] Arms L., Smith D.W., Flynn J., Palmer W., Martin A., Woldu A., Hua S. (2018). Advantages and Limitations of Current Techniques for Analyzing the Biodistribution of Nanoparticles. Front. Pharmacol..

[158] Croitoru G.-A., Pîrvulescu D.-C., Niculescu A.G., Grumezescu A.M., Antohi A.M., Nicolae C.-L. (2024). Metallic Nanomaterials—Targeted Drug Delivery Approaches for Improved Bioavailability, Reduced Side Toxicity, and Enhanced Patient Outcomes. Rom. J. Morphol. Embryol..

[159] Mu Q., Jiang G., Chen L., Zhou H., Fourches D., Tropsha A., Yan B. (2014). Chemical Basis of Interactions Between Engineered Nanoparticles and Biological Systems. Chem. Rev..

[160] Sengul A.B., Asmatulu E. (2020). Toxicity of Metal and Metal Oxide Nanoparticles: A Review. Environ. Chem. Lett..

[161] Jiao M., Zhang P., Meng J., Li Y., Liu C., Luo X., Gao M. (2018). Recent Advancements in Biocompatible Inorganic Nanoparticles towards Biomedical Applications. Biomater. Sci..

[162] Abd Ellah N.H., Abouelmagd S.A. (2017). Surface Functionalization of Polymeric Nanoparticles for Tumor Drug Delivery: Approaches and Challenges. Expert Opin. Drug Deliv..

[163] Frisch E., Clavier L., Belhamdi A., Vrana N.E., Lavalle P., Frisch B., Heurtault B., Gribova V. (2023). Preclinical In Vitro Evaluation of Implantable Materials: Conventional Approaches, New Models and Future Directions. Front. Bioeng. Biotechnol..

[164] Jeffers M.S., Xi C.E., Bapuji R., Wotherspoon H., Kimmelman J., Bedford P., McIsaac D.I., Lalu M.M., Fergusson D.A. (2024). Synthesizing Regulatory Guidance for Demonstrating Preclinical Efficacy and Translating Promising Cell Therapies to Early Phase Clinical Trials: A Scoping Review. BMC Med..

[165] Siafaka P.I., Üstündağ Okur N., Karavas E., Bikiaris D.N. (2016). Surface Modified Multifunctional and Stimuli Responsive Nanoparticles for Drug Targeting: Current Status and Uses. Int. J. Mol. Sci..

[166] Paliwal A., Jain S., Kumar S., Wal P., Khandai M., Khandige P.S., Sadananda V., Anwer M.K., Gulati M., Behl T. (2024). Predictive Modelling in Pharmacokinetics: From in-Silico Simulations to Personalized Medicine. Expert Opin. Drug Metab. Toxicol..

